# Path Models of Vocal Emotion Communication

**DOI:** 10.1371/journal.pone.0136675

**Published:** 2015-09-01

**Authors:** Tanja Bänziger, Georg Hosoya, Klaus R. Scherer

**Affiliations:** 1 Department of Psychology, Mid Sweden University, Östersund, Sweden; 2 Department of Educational Science and Psychology, Freie Universität, Berlin, Germany; 3 Swiss Centre for Affective Sciences, University of Geneva, Geneva, Switzerland; University of Sussex, UNITED KINGDOM

## Abstract

We propose to use a comprehensive path model of vocal emotion communication, encompassing encoding, transmission, and decoding processes, to empirically model data sets on emotion expression and recognition. The utility of the approach is demonstrated for two data sets from two different cultures and languages, based on corpora of vocal emotion enactment by professional actors and emotion inference by naïve listeners. Lens model equations, hierarchical regression, and multivariate path analysis are used to compare the relative contributions of objectively measured acoustic cues in the enacted expressions and subjective voice cues as perceived by listeners to the variance in emotion inference from vocal expressions for four emotion families (fear, anger, happiness, and sadness). While the results confirm the central role of arousal in vocal emotion communication, the utility of applying an extended path modeling framework is demonstrated by the identification of unique combinations of distal cues and proximal percepts carrying information about specific emotion families, independent of arousal. The statistical models generated show that more sophisticated acoustic parameters need to be developed to explain the distal underpinnings of subjective voice quality percepts that account for much of the variance in emotion inference, in particular voice instability and roughness. The general approach advocated here, as well as the specific results, open up new research strategies for work in psychology (specifically emotion and social perception research) and engineering and computer science (specifically research and development in the domain of affective computing, particularly on automatic emotion detection and synthetic emotion expression in avatars).

## Introduction

Accurately inferring the emotions of others in social interactions is extremely important, as it permits an understanding of the expresser's reaction to preceding events or behaviors and a prediction of the expresser's action tendencies and thus facilitates communication and interpersonal adjustment [1,2]. In consequence, the study of emotion expression and perception has become a major research area over the last 60 years and has played an important part in the development of emotion psychology as an interdisciplinary research area.

Emotions can be successfully communicated through vocal expressions alone (see reviews in [3–5]), but we still know little about the processes and mechanisms that allow humans to communicate emotions through vocal expressions [6]. In particular, the nature of voice characteristics (also referred to as vocal *cues* or vocal *features*) responsible for successfully expressing and recognizing emotions in vocal utterances are not yet well understood.

The study of nonverbal communication of emotion through voice and speech has been examined in past decades by focusing on either acoustic descriptions (e.g. [7–11]; and Table A in S1 File—Appendix) or recognition of emotions by listeners (e.g. [12–16]). Reviews of the field [3–5] often refer to these two approaches as *encoding studies*, focusing on the acoustic description of emotional vocalizations, and *decoding studies*, focusing on emotion recognition or discrimination by listeners.

A recent, comprehensive review of studies on facial, vocal, gestural, and multimodal emotion communication [5] calls attention to the following concerns: 1) Emotion expression (encoding) and emotion perception (decoding) are rarely studied in combination (and recognition studies are far more numerous than studies on the production of emotional expressions). As a consequence of the separation of these two central aspects of the communication process the underlying *mechanisms*, especially the nature of the cues used in emotion perception and inference, cannot be appropriately assessed. 2) Both encoding and decoding studies tend to focus on highly prototypical expressions of a handful of basic emotions. This raises important concerns: Prototypical expressions tend to increase the risk of stereotypical use of major emotion dimensions—especially *valence* (e.g., pleasantness-unpleasantness; [17]) in the case of facial expression and *arousal* in the case of vocal expression (see Table A in S1 File—Appendix). *Arousal* refers primarily to the physiological activation associated with emotional reactions and can be considered as a dimension ranging from intense activation to calmness or even sleep [18]. This bias, in addition to the few emotion alternatives generally provided for judgment in recognition studies, may lead to guessing and classification by exclusion in recognition studies ([5]; p. 415). Thus, the state of the art of research on vocal communication can be briefly characterized as follows: A handful of encoding studies shows that actors vocally enacting a relatively small number of basic emotions produce differentiated patterns of vocal parameters for different emotions (with a preponderance of arousal-related parameters). A rather large number of decoding or recognition studies shows that naïve judges recognize portrayals of a relatively small number of basic emotions with better than chance accuracy (although effects of guessing and classification by exclusion, based on arousal cues, cannot be excluded). Therefore a more comprehensive, integrative approach is needed to advance research on the mechanisms underlying the vocal communication process.

Here we examine the utility and feasibility of studying the encoding, transmission, and decoding phases of the vocal emotion communication process by using a *Brunswikian lens model* approach which is particularly well suited for this purpose as it allows combining encoding and decoding processes. In particular, we show that comprehensive models and their quantitative testing provide an important impetus for future research in this area, not only by providing a more theoretically adequate framework that allows hypothesis testing and accumulation of results, but also by pointing to areas where further method development is urgently required (e.g. the development of reliable measurement for new acoustic parameters that can be expected to correlate with voice quality perception).

We first describe the general framework provided by the lens model (with a focus on the variants of the model that are used for the analysis presented in this article). We then outline the statistical models that can be used for empirical model testing.

### Theoretical models–from Brunswik's lens model to the TEEP

Brunswik [19] proposed that successful adjustment to an uncertain, constantly changing world requires the organism to rely on probabilistic inference mechanisms using multiple pieces of uncertain evidence (proximal cues) about the world (the distal object). He illustrated this process by a *lens-shaped model* in which a fan-shaped array of probabilistic sensory cues for a distal object are utilized to form a singular judgment about the object. The fit of this subjective judgment with world reality he called *ecological validity*. Brunswik originally focused on visual perception, but already proposed several variants of his lens model applied to the study of interpersonal perception. The simplest version of the lens model in this domain includes (a) a distal “object” (when applied to vocal communication of emotion, the emotion experienced by the speaker), (b) a number of observable and measurable cues (the vocal features affected by the emotion and used by the listener to infer the emotion), and (c) a perception or perceptual judgment from a human observer (the emotional attributions made by the listener). Examples of studies of interpersonal perception that make explicit reference to the lens model include analysis of the nonverbal communication of interpersonal dispositions [20], perception of intelligence from audio-video recording [21], perceived “quality of relationship” [22], and several recent studies on personality expression and inference, such as self-other agreement at zero acquaintance [23], hindsight effects and knowledge updating [24], the perception of trustworthiness from faces [25], behavioral cues of overconfidence [26], and vocal cues of hierarchical rank [27]. Juslin and his collaborators have used this functional approach to cue utilization in studying emotion communication in music performances [28–31] and for the encoding and decoding of vocal emotions [32].

In an early study on the expression and perception of personality in the speaking voice, Scherer [33] proposed and tested an extension of the lens model in which the cue domain is separated into (a) *distal*, *objectively measurable cues* (such as acoustic voice parameters for the speaker) and (b) *subjective*, *proximal percepts* of these cues (such as voice quality impressions formed by the listener). The major justification for this extension is that in perception and communication, the objectively measurable cues in nonverbal behavior are subject to a transmission process from sender to receiver (often adding noise) and need to be processed and adequately transformed by the sensorium of the receiver. A comprehensive model of the communication process requires conceptualization and empirical measurement of this transmission process ([4]; see also [34, 35]).

**Fig 1:**
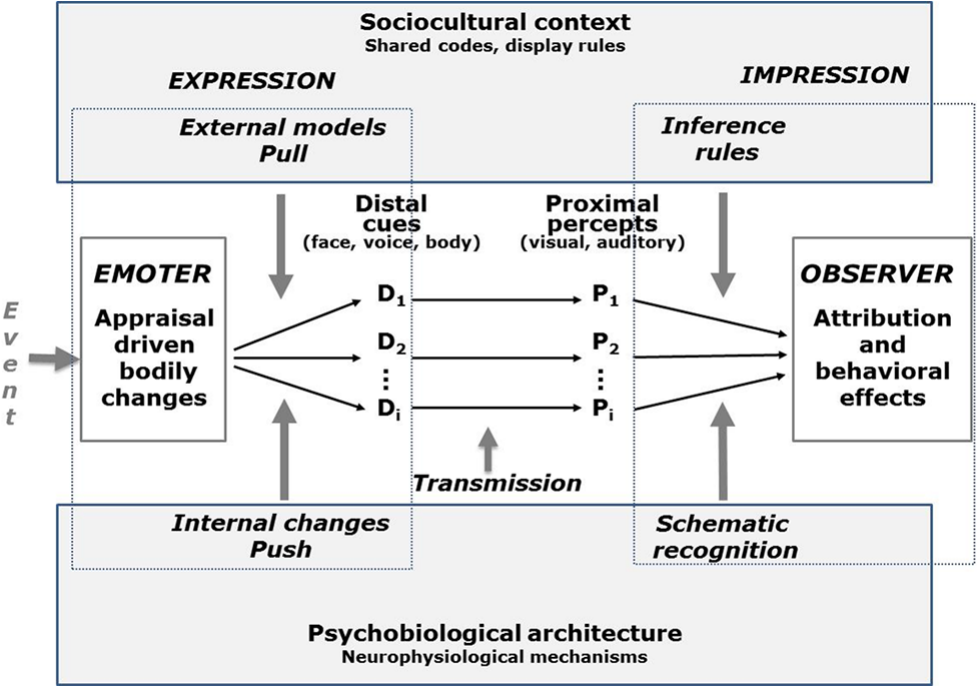
The tripartite emotion expression and perception (TEEP) model (based on Brunswik's lens model). The terms “push” and “pull” refer to the internal and the external determinants of the emotional expression, respectively, distinguished in the lower and upper parts of the figure. D = distal cues; P = percepts. Adapted from p. 120 in Scherer [36].

More recently, Scherer [36] has formalized the earlier suggestion for an extension of the lens model as a *tripartite emotion expression and perception* (TEEP) model (see Fig 1). The communication process is represented by four elements (emoter/sender, distal cues, proximal percepts and observer) and three phases (externalization driven by external models and internal changes, transmission, cue utilization driven by inference rules and schematic recognition). Applying this model to our specific research questions, the internal state of the speaker (e.g. the emotion process) is encoded via *distal* vocal cues (measured by acoustic analysis); the listener perceives the vocal utterance and extracts a number of *proximal* cues (measured by subjective voice quality ratings obtained from naive observers); and, finally, some of these proximal cues are used by the listener to infer the internal state of the speaker based on schematic recognition or explicit inference rules (measured by naive observers asked to recognize the underlying emotion). The first step in this process is called the *externalization* of the internal emotional state, the second step the *transmission* of the acoustic information and the forming of a *perceptual representation* of the physical speech/voice signal, and the third and last step the *inferential utilization* and the emergence of an emotional attribution.

Next we describe the statistical models that have been used in earlier studies for Brunswikian lens modeling, with a focus on the two models that are used in the empirical part of the present article.

### Statistical Paradigms for Lens Modeling

**Fig 2:**
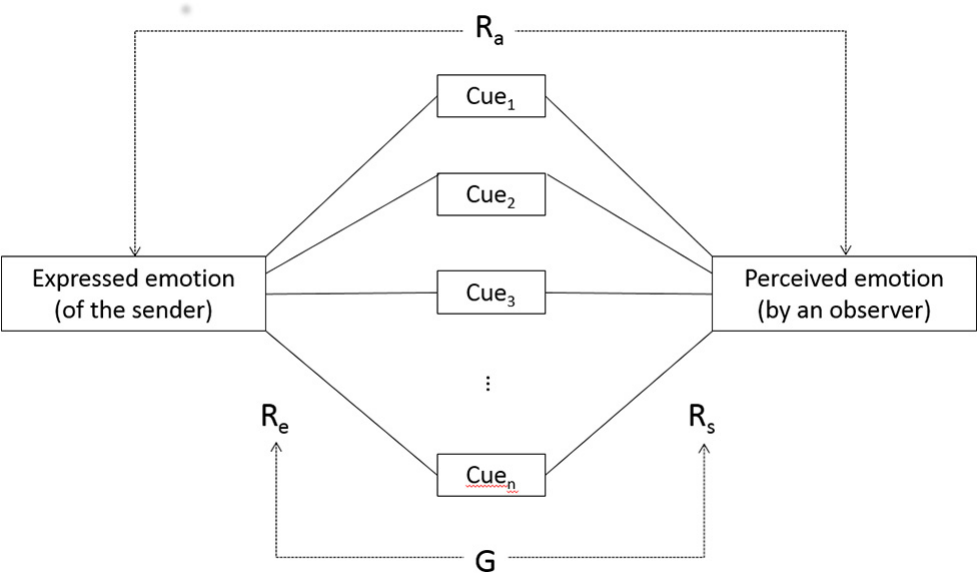
Graphic illustration for the Lens Model Equation.

The dominant statistical paradigm in work informed by a Brunswikian approach is the *lens model equation* (LME [37]), originally developed by Hammond, Hursch, and Todd [38] and Tucker [39]. The LME is essentially based on two regression equations and two correlations. Fig 2 provides a graphical illustration adapted to the vocal communication of emotion. In a first regression equation, objectively measurable cues are predictors for the distal criterion (*expressed emotion* in Fig 2). The corresponding multiple correlations (R_e_) on the left side of the graph represent the ecological validity (i.e. the extent to which the measured cues account for the variance in the distal criterion). The second regression equation uses the same cues as predictors for the proximal judgments of an individual with regard to the distal criterion. The corresponding multiple correlation (R_s_) on the right side of the graph indicate the extent to which the cues in the model can account for the listeners’ attributions (cue utilization). The weights of individual cues in the regressions are not part of the LME itself, but are sometimes considered in order to investigate the independent contribution of various cues in the models (both with respect to ecological validity and with respect to cue utilization). A correlation coefficient (between criterion and judgment) is used to represent *accuracy* (R_a_ in Fig 2). Another correlation coefficient is used to assess the correspondence between the two regressions (G in Fig 2). Juslin and collaborators [28,30,31] have adopted this paradigm in their work on emotional communication in music performances.

**Fig 3:**
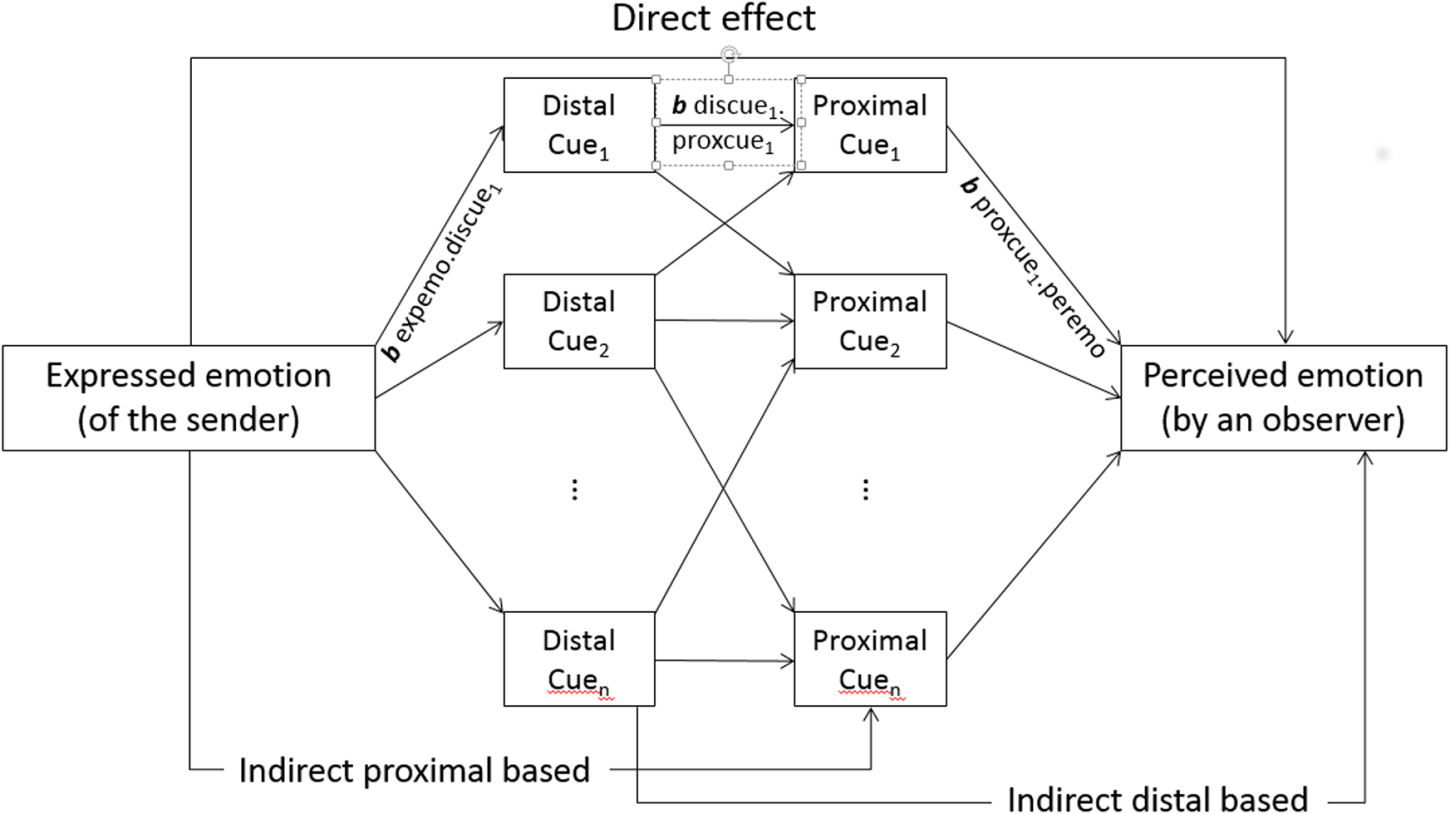
Graphic illustration for an extended model (path analysis with separate distal and proximal cues).

In Scherer’s [33] extension of the Brunswikian lens model (in his work on the expression and perception of personality in vocal communication), an early version of path analysis (as proposed by Duncan [40]) was used (see Fig 3) in which the accuracy coefficient (i.e., the correlation between expressed and perceived emotion) can be split into (a) the contributions of the postulated central indirect paths, (b) peripheral indirect paths (either distally based, bypassing the percept component, or proximally based, bypassing the distal cue component), and (c) the remaining direct path (i.e., the variance explained that is not accounted for by the mediation).

The present article describes a first attempt to demonstrate the plausibility of the model assumptions by examining how well the model can account for empirical data on the emotion expression in the voice (*externalization*) and the corresponding inferences made by naive observers (*utilization*). An ancillary question that has hardly been addressed in the literature concerns *cue transmission*—the degree to which the proximal cues appropriately reflect emotion-differentiating distal cues and what the nature of the mapping is. For this purpose, we examined the respective contributions of the LME (Fig 2) and the statistical model derived from the TEEP (path analysis illustrated in Fig 3) in a re-analysis of two corpora of vocal emotion portrayals, using an exploratory approach. Specifically, we attempt to empirically determine the relative importance of different variables and their associations rather than testing specific hypotheses.

The data used for the re-analyses have been collected in two consecutive research programs with different corpora of vocal expressions enacted by professional actors using Stanislavski techniques (reconstituting vivid feelings by recalling past emotional experiences; [41]). The results reported here are the product of studies conducted over a period of 15 years, during which the two corpora were recorded with professional actors (the “Munich” corpus [MUC] [9]; and the “Geneva” corpus [GVA], Geneva Multimodal Emotion Portrayals—GEMEP [42]); appropriate stimuli were selected for ground truth and authenticity [43]; acoustic analyses were developed, applied, and validated [9,10]; and a new subjective voice rating scale was developed and validated [44]. It was only after this preliminary work that all the necessary elements were available to proceed to an overall modeling of the TEEP model applied to vocal emotion communication. Although some of the raw data here have been used for earlier reports on development and validation, so far there has been no attempt to link the expression, or encoding, side to the inferential, or decoding, side using both distal acoustic parameters and subjective proximal ratings as mediators. In consequence, the analyses and results presented here are original to the current article.

## Methods

### Description of the Corpora Used in the Analyses

Detailed descriptions of speech recording, analysis and ratings are described in [42] and [44]. In consequence, we limit the description of the methods to an overview of the procedures that are essential for understanding the main features of the data used for the Brunswikian re-analysis (further details can be found in the original papers or in the supplementary information in S1 File–Appendix).

### Selection of Emotion Portrayals

The recordings of emotion portrayals used from the MUC corpus were produced by German-speaking professional actors who enacted all emotions while articulating two meaningless pseudo-speech sentences (without any semantic content): (a) “hät san dig prong nju ven tsi” and (b) “fi gött laich jean kill gos terr” [9]. For the current analyses, 144 expressions from this corpus have been used, corresponding to 16 portrayals produced by nine actors (four men and five women) for eight emotions (hot and cold anger, elation and calm joy, despair and sadness, panic fear and anxiety). Each pair of emotions listed corresponds to one family (anger, happiness, sadness, and fear). The first member of the pair is defined as involving high emotional arousal, whereas the second member of each pair involves low emotional arousal.

The recordings used from the GVA corpus were produced by French-speaking actors who enacted all emotions, coached by a professional director, while articulating two meaningless pseudo-speech sentences: (a) “ne kal ibam soud molen” and (b) “koun se mina lod belam” [44]. The eight emotions with the closest possible match to those in the MUC corpus were chosen. The GVA corpus was recorded to include emotions equivalent to those that were used in the MUC corpus. Different labels were used because the actors/encoders producing the portrayals in both corpora spoke different languages (German for MUC and French for GVA), but essentially the definitions of emotions used were similar, with the exception of “pleasure” (“plaisir” in French) and “calm joy” (“Stille Freude” in German), which were not defined as corresponding to identical states, but which were nevertheless both intended to be positive emotions with low arousal. From the GVA corpus, 160 expressions were used, corresponding to 10 actors (five women) who portrayed the eight emotions by using the two pseudo-speech sentences.

### Objective Acoustic Measures (Distal Cues)

Distal voice cues are general assessed via objective acoustic measurement of vocalizations. Given the complexity of this domain we cannot describe the measures and procedures in detail (see [6, 9, 10] for more extensive discussions). Parameter extraction for both corpora was performed with the open source speech analysis program PRAAT [45]. The extraction procedures are described in [44] (methodological details on the acoustic extractions are also provided in S1 File—Appendix). Acoustic parameters to be extracted for the MUC corpus were chosen from the extensive list in Banse and Scherer [9]. Two of these measures (spectral slope and jitter) were excluded after extraction based on the assessment of reliability carried out for all measures. As the 44 extracted parameters extracted showed a high degree of collinearity (in the MUC corpus) a principal component analysis was calculated in order to select a reduced number of acoustic parameters. This analysis showed that nine factors allowed accounting for 80% of the variance present in these data. The full results of the PCA are available in Tables B-D in S1 File—Appendix. Nine parameters were selected (one for each factor in the analysis; see Table D in S1 File—Appendix). Acoustic intensity did not constitute an independent factor in the analysis, but given its importance for emotion expression and communication, the parameter "mean intensity" was added to this list. Two initially selected parameters did not differentiate the expressed emotions and were therefore discarded. The acoustic parameters included in the present analyses are shown in Table 1, indicators of fundamental frequency (F0), acoustic intensity, duration of speech segments (tempo) and measures of spectral energy distribution. As formant analyses were not carried out on the recordings of the MUC corpus, articulatory effects could not be assessed.

**Table 1:** Eight acoustic parameters selected for the LME analyses.

Domain	Description	Label
Fundamental frequency (F0)	Minimum or 5^th^ percentile of the F0 represents the floor/level of the fundamental frequency.	F0 floor / F0 5th percentile^a^
	Range (difference between minimum and maximum) represents the variability of the fundamental frequency.	F0 range
Intensity	Mean represents the acoustic intensity level.	Intensity mean
	Range (difference between minimum and maximum) represents the variability in acoustic intensity.	Intensity range
Duration	Total duration (of the utterance) represents the speech rate (all utterances have the same number of syllables).	Acoustic duration
	Relative duration of voiced segments on speech segments (duration of voiced divided by the sum of the duration of voiced and unvoiced segments, i.e. excluding phonetic interruptions) represents the relative duration of voiced speech segments (i.e. a variable related to accentuation; prolonged vocals reflect more accentuated speech).	Relative duration
Distribution of energy in the long-term averaged spectrum (voiced segments only)	0–1000 Hz (relative to 0–8000 Hz) represents the proportion of spectral energy in “low” frequency regions (i.e. a variable that reflects voice quality—a sharp voice is characterized by increased energy in the higher frequency regions).	Relative energy <1000
	600–800 Hz (relative to 0–8000 Hz) represents voice quality changes; this variable was selected because it was only mildly correlated with LTSv < 1000, and it loaded on an independent factor in the PCA computed on all acoustic variables extracted on the MUC corpus.	Relative energy <800

LME = lens model equation; LTSv = long-term averaged spectrum (voiced segments); PCA = principal component analysis.

^a^ For the MUC corpus, the F0 contours were manually corrected (for extraction mistakes, such as octave jumps and detection of periodicity in unvoiced segments). For the GVA corpus, no such corrections were made. Consequently, the absolute minimum of F0 detected in each utterance was used for the MUC corpus, whereas the 5^th^ percentile of the automatically extracted F0 was used for the GVA corpus.

As there is sizeable and systematic variation in acoustic features across speakers (e.g. female voices having much higher fundamental frequency than male voices) all acoustic parameters were standardized within speaker (for both corpora) to control for these extraneous sources of variance.

The parameters listed in Table 1 were used for the LME analyses. The same set of parameters was used for the computation of the path analyses, except for two parameters which were excluded to reduce the number of variables to be included in the models: the relative duration of voiced segments and the proportion of energy between 600 and 800 Hz (chosen because these rarely used parameters partly overlapped with other parameters and thus did not provide incremental contributions to the variance in the LME analyses).

### Subjective Voice Quality Ratings (Proximal Percepts)

The procedures used to collect ratings of proximal voice cues have been described and justified in detail in Bänziger et al. [44] (see also S1 File—Appendix). Here we describe only the essential aspects needed to understand and interpret the models we present in the current article.

Several groups of participants were recruited to assess the proximal voice cues in successive rating studies for both corpora. All ratings were collected in small laboratories dedicated to perception/judgment studies at the University of Geneva. Individual computers and closed-ear headphones were used to present the vocal portrayals, and computer interfaces were created to record the raters’ answers. The raters were all students at the University of Geneva and were compensated for their services, either in the form of course credit or financial remuneration. Average ratings are used for the analyses presented in the current paper. The averages were obtained from 61 raters (48 women, average age 21 years) for the MUC corpus, but with only 15 or 16 ratings for each stimulus. Different raters provided ratings for various subsets of the corpus. For the GVA corpus, 19 participants (10 women, average age 22 years) provided ratings for all scales. Further details on the rating procedures have been published in [44]. Table E in S1 File—Appendix provides the details of the composition of the various groups of raters involved in rating proximal voice cues in both corpora and displays the estimates of inter-rater reliabilities obtained for the various ratings. The level of reliability of the ratings ranged from very good to satisfactory (all Cronbach's alpha values were larger than .80). The proximal voice scales to be rated were selected in a series of pilot studies designed to identify vocal dimensions that could be assessed by untrained raters with acceptable consistency. Eight vocal dimensions were chosen to be included in the Geneva Voice Perception Scale (GVPS; see [44]) and were used for both corpora described in this article. The eight scales are shown in Table 2.

**Table 2:** Scales used for the voice ratings, translations, and terms used in the study with French-speaking raters.

English translation		French scale names (used in the study)	
Scale	Direction	Scale	Direction
Pitch	low ↔ high	Hauteur	grave ↔ aiguë
Loudness	soft ↔ loud	Volume	faible ↔ forte
Intonation	monotonous ↔ accentuated	Mélodie	monotone ↔ modulée
Speech rate	slow ↔ fast	Vitesse	lente ↔ rapide
Articulation	poor ↔ good articulation	articulation	mal ↔ bien articulée
Instability	steady ↔ trembling	stabilité	ferme ↔ tremblante
Roughness	not rough ↔ rough	qualité rauque	non rauqe ↔ rauque
Sharpness	not sharp ↔ sharp	qualité perçante	non perçante ↔ perçante

The GVPS was used for the ratings in both corpora, but the procedures involved in collecting the ratings differed slightly. For the MUC corpus, the perceived voice ratings were collected by a stimulus ranking procedure of the emotion portrayals, separately for each speaker. On a computer screen, raters arranged icons representing the audio stimuli (which they could listen to repeatedly) recursively on a continuum that was consequently recoded to a score ranging from 0 to 100. For the corpus, a more conventional rating procedure was involved, with raters using a visual analogue scale on the screen immediately after listening to each portrayal (later recoded to a score ranging from 0 to 100). All participants provided ratings for all voice scales sequentially and in random order (stimuli were also presented in random order for assessment within each scale).

### Assessment of Perceived (Attributed) Emotions

For the MUC corpus, the perceived (attributed) emotions for each stimulus were assessed by asking groups of raters to judge the perceived intensity of fear, anger, happiness, and sadness by using the recursive stimulus ranking procedure described earlier for the ratings of perceived voice cues. The ratings were provided by 56 participants (45 women, average age 22 years). Different raters provided ratings for various subsets of the corpus; 14 ratings were collected for each stimulus. The ratings were made on visual analogue scales and the answers were rescaled to vary between 0 and 100 (0 = *the emotion is absent*, 100 = *extreme emotional intensity*).

For the GVA corpus, emotion recognition accuracy was assessed by asking 23 raters (13 women, average age 29 years) to listen to all emotional expressions included in the larger database (in random order but grouped for each speaker) and to produce a categorical rating (selection of one emotional category among 18 alternatives, or no emotion present) and an intensity rating for each portrayal (the procedure and detailed results have been reported in [44]; see also S1 File—Appendix). Recognition accuracy estimates were computed as the proportion of raters providing an accurate answer (i.e. selecting the emotion category matching the expressive intention of the actor). Arcsine transformations were then applied to the proportional emotion recognition scores (resulting in a score between 0 and 100). None of the raters assessing emotions participated in the assessment of the GVPS (i.e. ratings on emotions and GVPS are obtained independently for both corpora). Table E in S1 File—Appendix provides information on the groups of raters involved and the estimated inter-rater reliabilities. Reliabilities ranged from very good to satisfactory (all alpha values larger than .80).

### Assessment of Perceived Emotional Arousal

One of the aims of the present analyses was to examine the role of arousal in the vocal communication process. In consequence, we obtained ratings of the arousal manifested by the speakers. For the MUC corpus, a separate group of 24 raters (all women, 22 years old on average; not involved in the ratings of emotions or GVPS) assessed the degree of perceived emotional arousal in all portrayals, using the recursive stimulus ranking procedure described earlier for the GVPS.

For the GVA corpus, the proximal voice ratings (GVPS) and the arousal ratings were obtained from the same 19 raters. After providing the ratings for the eight voice scales, the emotional expressions were presented again (in a new random order for each rater), and the raters had to assess arousal on a visual analogue scale presented on the screen. Table E in S1 File—Appendix provides the information on the raters involved and the estimated inter-rater reliability which was equally large in both corpora (alpha = .98).

The results showed that the ratings obtained for the two different data sets with the different rating procedures were remarkably similar [44]. In the analyses reported in the following sections, we used aggregated scores in the form of the mean values reported for each portrayal on each rating scale (GVPS; emotional intensity/accuracy and arousal ratings). For the path analyses, we standardized the average ratings obtained on the GVPS.

### Methods of Statistical Modeling

As described in the introduction, we used two different approaches for modeling: (a) the Brunswikian LME and (b) path analysis with both distal and proximal cues into a single model (as shown in the TEEP model; Fig 1). For ease of comprehension, we provide the details on the modeling paradigms and the statistical operations in the Results section before the description of the results, separately for each of the two approaches.

### Ethical statement

This work has been performed under strict observance of the ethical guidelines elaborated by the Ethics Committee of the Department of Psychology at the University of Geneva. Specifically, the Ethics Committee requested that we submit a detailed description of all studies to be conducted in the research program “Production and perception of emotion” funded by the European Research Council (ERC). We described all procedures in detail and confirmed that we would follow the instructions of the Ethics Committee concerning the procedure to obtain informed consent. Based on this declaration, the procedures in the series of studies were approved. For the professional actors we obtained informed consent to produce the required emotion enactments and that we could use these for research purposes. The remaining participants produced only ratings of the actor-expressed emotions. In consequence, they were not subject to any experimental manipulation. Raters were recruited from the student body of the University of Geneva via posted announcements describing the aim of the study and the procedures used for the ratings. They recorded their agreement to produce the ratings against payment or course credit on enrollment sheets which provided a detailed description of the rating procedure. All raters were informed of their right to abandon their rating activity at any time. Raters choosing to be paid recorded their consent to have their data used for research purposes by their signature on a form sheet that also served to document payment received for the ratings. Raters choosing to obtain course credit signed a consent form that stipulated that the data would be stored anonymously and course credit was granted based on the enrollment sheets specifying their choice of compensation (names were registered separately of the data recorded during the study).

In all cases, age, gender and native language of the raters were recorded along with the data collected during the rating sessions. The students were also required to report if they had any form of diagnosed deficit in auditory perception (without having to provide any further details; their reply to this question was recorded along with their ratings, anonymously).

It should be noted that some actor recordings and some rating studies for the MUC corpus were performed before the existence of an ethics committee in the Department of Psychology at the University of Geneva. However, the procedures used were identical to those later approved by the current ethics committee.

## Results

### LME Modeling

The LME ([39, 46]; see Eq 1 and Fig 2) computes *communication achievement* (*r*_*a*_, i.e. the correlation between the expressed and perceived emotion) as the sum of two components: the *linear component* (i.e. the component of the correlation derived from the linear contributions of the variables entered in the model) and the *unmodeled component* (which includes systematic and unsystematic variance not accounted for by the linear component). The linear component is a product of *speaker consistency* (*R*_*e*_, which corresponds to the multiple correlation of enacted emotion on the variables in the model), *rater consistency* (*R*_*s*_, i.e. the multiple correlation of perceived emotion on the variables in the model), and *matching* (*G*, i.e. the correlation between the predicted values of the expressed emotion model and the predicted values of the perceived emotion model).

$$r_{a}=G\times R_{e}\times R_{s}+C\times \sqrt{1-R_{e}{}^{2}}\times \sqrt{1-R_{s}{}^{2}}$$ (1)

The unmodeled component is the product of three parameters represented in the second term of the addition in Eq 1. Parameter *C* corresponds to the correlation between the residuals of the two multiple regressions, it can be derived from the values of the other parameters of the equation that are reported in the result Tables for the LME. In this model, a value close to 1 for parameter *G* indicates a good match in terms of the use of vocal features on the two sides of the model. In contrast, a value close to 0 for this parameter indicates that the use of vocal features is different for encoding and decoding. Low values (approaching zero) for the parameters *R*_*e*_ and *R*_*s*_ may be the consequence of several factors that the model does not allow considering separately: (a) The vocal features in the model are used inconsistently; (b) the vocal features in the model are used in a nonlinear way (i.e. nonlinear functions of these features might allow prediction of the emotion enacted and the emotion perceived); (c) the vocal features important for encoding or decoding are not included in the model; and (d) the measurement errors are large for the variables considered.

This model provides indices that are essentially descriptive. All indices are correlations or multiple correlations and therefore represent effect sizes (proportion of variance shared/explained between the respective variables). In the result tables, we include the ratio of (*G* × *R*_*e*_ × *R*_*s*_)/*r*_*a*_. This ratio represents the proportion of the relationship between the expressed and perceived emotion that is accounted for by the voice features included in the model. All regression coefficients for the LME analyses including levels of significance are provided in Table A of S2 File—Data.

We computed separate LMEs for each corpus, for each of the four emotion families, and for distal cues and proximal percepts. The results for both corpora and all emotion families and arousal are shown in Table 3 (for both *distal cues* and *proximal percepts*). These tables include the parameters that compose the *linear component* of the LME: *achievement* (*r*_*a*_, the correlation between emotion enacted and emotion perceived), *ecological validity* (*R*_*e*_, the multiple correlation between the acoustic parameters or the perceived vocal features, and the emotion enacted), *functional validity* (*R*_*s*_, the multiple correlation between the acoustic parameters or the perceived vocal features, and the emotion perceived), *matching* (*G*, the correlation between the variables predicted derived from the regression of the acoustic parameters or the perceived vocal features on the emotion enacted and on the emotion perceived), and the ratio of (*G* × *R*_*e*_ × *R*_*s*_)/*r*_*a*_, which represents the proportion of the relationship between the expressed and perceived emotion that is accounted for by the respective voice features included in the model. Fig A in S2 File—Data illustrates the case of anger. The standardized beta coefficients for the regressions obtained for these models are reported in Table A of S2 File—Data.

**Table 3:** Summary of five LMEs (four emotion families and arousal) for both corpora based on eight acoustic parameters (same parameters used in all models) or eight averaged voice ratings (same voice scales in all models).

Emotion families and arousal	Corpus	*r*_*a*_	*R*_*e*_	*R*_*s*_	*G*	*R*_*e*_×*R*_*s*_×*G*/*r*_*a*_
		Achievement	Ecological validity	Functional validity	Matching	
**Models based on eight acoustic parameters (distal cues)**						
anger	MUC	.764	.702	.763	.828	0.58
	GVA	.843	.501	.652	.949	0.37
fear	MUC	.670	.558	.598	.826	0.41
	GVA	.784	.455	.545	.937	0.30
happiness	MUC	.735	.238	.365	.304	0.04
	GVA	.896	.498	.514	.943	0.27
sadness	MUC	.774	.549	.670	.896	0.43
	GVA	.786	.380	.388	.926	0.17
arousal	MUC	.723	.776	.891	.953	0.91
	GVA	.860	.891	.952	.988	0.97
**Models based on eight perceived voice cues (proximal cues)**						
anger	MUC	.764	.756	.858	.841	0.71
	GVA	.843	.799	.870	.948	0.78
fear	MUC	.670	.582	.773	.788	0.53
	GVA	.784	.617	.751	.942	0.56
happiness	MUC	.735	.494	.686	.775	0.36
	GVA	.896	.541	.598	.977	0.35
sadness	MUC	.774	.680	.829	.956	0.70
	GVA	.786	.448	.631	.953	0.34
arousal	MUC	.723	.812	.961	.927	1.00
	GVA	.860	.897	.965	.971	0.98

The data shown in Table 3 allow several types of comparison. The most important information is the proportion of the relationship between the emotion family expressed and the emotion family inferred (“achievement,” shown as the correlation between these two variables in the first column) that is accounted for by the mediating variables in the model (this proportion is displayed in the last column of Table 3). While the values for achievement are the same, the values for the proportion accounted for show major differences for the distal and the proximal models. In all cases except for arousal, where both models perform about evenly, the proximal model explains more variance than the distal model, in some cases accounting for almost double the variance. This discrepancy cannot be accounted for by a lower level of matching between expression and inference, as index *G* is quite comparable for both models. In other words, the respective cues, distal or proximal, are appropriately used in inference, in line with the information they contain. Rather, the comparison of the models shows, on average, both lower ecological validity (i.e. captures less distinctiveness among the emotions expressed) and lower functional validity (i.e. contributes less to the variance in the inference) for distal cues than for proximal cues. This discrepancy seems somewhat more pronounced in the case of GVA corpus for anger, fear, and sadness. It is unlikely that the expressions in this corpus carry less acoustic information, since there are no such differences in the proportion accounted for between the corpora for the proximal model (except in the case of sadness), which suggests that the information is available and is correctly interpreted. Methodological issues can be excluded, as the same acoustic parameters were used, calculated with the same software. However as a relatively high amount of statistical error variance cannot be ruled out, any further attempts at interpretation seem moot.

The results observed for the arousal ratings shown in Table 3 are highly similar in both corpora and strongly corroborate the dominant role of arousal in vocal emotion communication. For both the distal and proximal models, the parameters show almost complete explanation of the achievement by the respective variables. In other words, arousal differences in vocal emotion expressions are well captured by acoustic variables and voice ratings and play a powerful role in the inference by listeners.

Apart from some level differences, the values for the two corpora were highly comparable (profile correlations on the LME parameters are *r* = .55 for the models based on distal cues and *r* = .72 for models based on proximal cues). Kolmogorov-Smirnov-Tests were computed for all variables over the two datasets to test the equality of the probability distributions. All of the tests yielded statistically non-significant results. In consequence, it was decided to combine the two data sets for the following analyses, which include both distal and proximal cues, as the statistical tests of the TEEP model with path analyses requires more observations to obtain sufficient statistical power, given the larger number of variables and covariates.

### Path Analysis Based on the TEEP Model

We adopted the path analysis approach described by Scherer [33] (based on [40]) to model the vocal communication of emotion for the merged corpora with a total of 304 vocal emotion portrayals. It should be noted that even the pooled sample size is still low with respect to the number of parameters to be estimated in the path model (df = 204). Lei and Wu [47] recommend a minimum of 5 cases per estimated parameter. In the path model described below, we used expressed happiness as a reference category (a separate analysis for happiness, compared with the other expressed emotions, can be found in Tables D and E in S2 File—Data).

**Fig 4:**
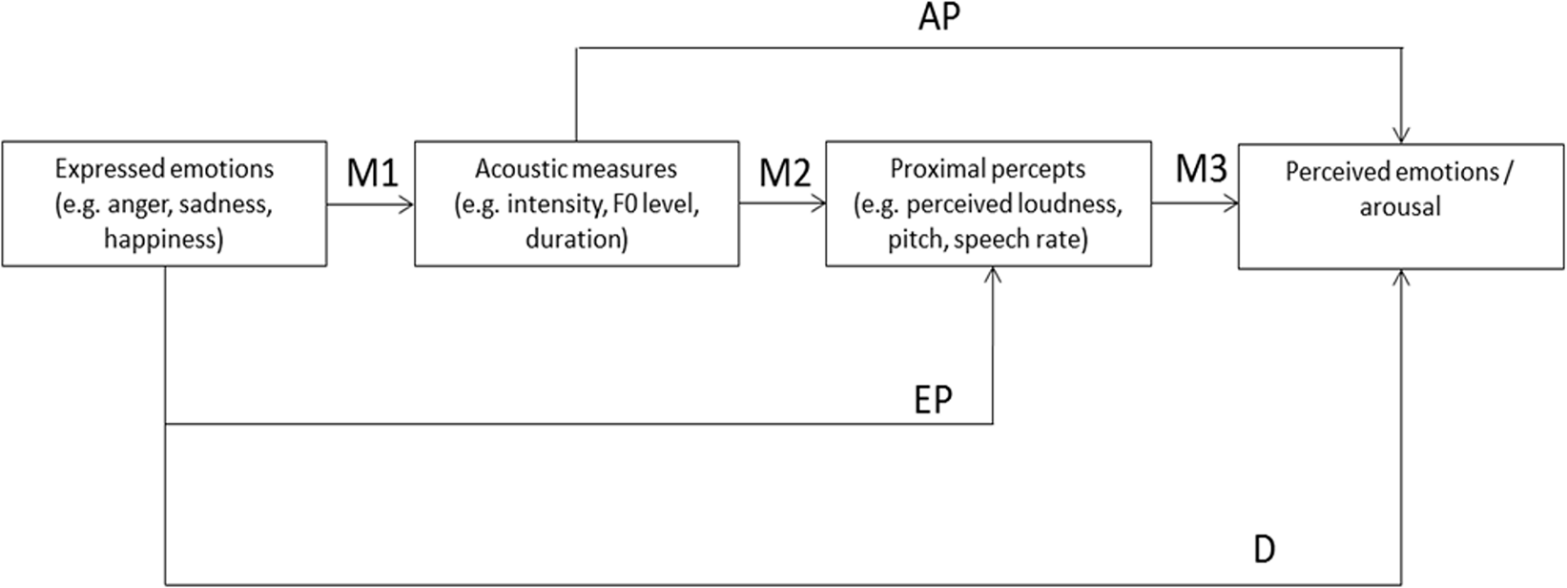
Conceptual representation of the TEEP path model.

Fig 4 illustrates the conceptualization of the TEEP path model for the current analysis. The leftmost box, labeled “expressed emotions,” represents the binary coded emotions enacted by the actors (as well as the operationally defined expressed level of arousal).

The second box, labeled “acoustic measures”, represents the extracted acoustic characteristics (i.e. the distal cues in the TEEP). The *z*-standardized acoustic cues used are mean intensity, intensity range, F0 floor 5^th^ percentile, F0 range, acoustic duration, and relative energy < 1000 (see Table 1).

The third box represents the perceived characteristics of the vocal portrayals (i.e. the proximal percepts in the TEEP model), consisting of the *z*-standardized voice quality ratings: intonation, loudness, pitch, roughness, speech rate, and instability. The rightmost box represents the perceived emotion(s) and the perceived arousal level. An arcus-sinus-square root transformation was applied to these variables, which were originally bound between 0 and 1.

The arrows in Fig 4 show the effects that were included in the model: (a) the direct path from the expressed to the perceived emotion (D); (b) paths from the expressed emotions to the proximal percepts bypassing the acoustic measures (EP); (c) paths from the acoustic measures to the perceived emotions bypassing the proximal percepts (AP); and (d) all paths via both the distal and the proximal cues (M1 to M3). The path group M1 allows one to assess how a certain emotional state of the sender is encoded into objectively measurable acoustic parameters, the path group M2 allows assessment of how the physical characteristics of the voice signal are translated into proximal percepts (transmission), and the last path group M3 is indicative of how the proximal percepts are used to infer an emotional state of the sender from the signal (decoding). Clearly, perfect mediation in the TEEP model would be indicated by all effects passing from M1 to M3 with no effects for the paths AP, EP, and particularly for the direct path D. In addition to analyzing all direct and indirect paths, we estimated covariances between all dependent variables belonging to the same variable group (acoustic measures, proximal percepts, perceived emotions) with the software Mplus [48], using the estimation procedure with robust standard errors. The input instructions for the model are documented in Table B and excerpts of the output in Table C in S2 File—Data.

As the model is fairly large, the results are presented in three separate tables. Only paths reaching a significance level of *p* < .02 are reported to guard against overinterpretation. These significant paths are also illustrated by a series of separate figures (one for each negative emotion and one for arousal), in order to facilitate interpretation. Table 4 shows the path groups M3, AP, and D (as illustrated in Fig 4) with the perceived emotions as dependent variables. The path group M3 allows assessment of cue utilization in the Brunswikian sense. For example, the detection of anger is predicted by perceived loudness (*b* = .296), low perceived instability (*b* = -.257), and high roughness (*b* = .177). The path group AP allows assessment of the contribution of the acoustic measures to the detection of anger. The results indicate that the acoustic measures included in the model contribute only to the prediction of perceived arousal. Finally, the path group D indicates the direct effects from expressed emotions to perceived emotions. For example, perceived anger is predicted by expressed anger (*b* = .566) and expressed fear (*b* = .145). The high path coefficients for expressed anger as a predictor indicates that not all information regarding the expressed emotion is mediated through the acoustic measures and the proximal percepts. The high path coefficients for expressed fear indicates that it may sometimes mistakenly be identified as anger.

**Table 4:** Prediction of *perceived emotions* by proximal percepts, distal cues, and expressed emotions, including standardized partial regression coefficients, *R* ^2^, and incremental *R* ^2^.

Perceived emotion (DV)	Significant predictors (p = < .02)	Standardized partial regression coefficient b	R2	ΔR2
Perceived anger	Proximal percepts (M3)		.638***	
	Loudness	.296**		
	Instability	-.257***		
	Roughness	.177***		
	Distal cues (AP)		.655***	.017**
	Expressed emotion (D)		.762***	.107***
	Expressed anger	.566***		
	Expressed fear	.145**		
Perceived fear	Proximal percepts (M3)		.487***	
	Speech rate	.168**		
	Instability	.356***		
	Distal cues (AP)		.521***	.034***
	Expressed emotion (D)		.713***	.192***
	Expressed fear	.668***		
	Expressed anger	.130*		
	Expressed sadness	.257***		
Perceived happiness	Proximal percepts (M3)		.265***	
	Intonation	.205**		
	Distal cues (AP)		.269***	.004
	Expressed emotions (D)		.620***	.351***
	Expressed anger	-.717***		
	Expressed sadness	-.654***		
	Expressed fear	-.652***		
Perceived sadness	Proximal percepts (M3)		.414***	
	Intonation	-.208**		
	Loudness	.284*		
	Speech rate	-.211**		
	Instability	.279***		
	Distal cues (AP)		.450***	.036***
	Expressed emotions (D)		.660***	.210***
	Expressed sadness	.630***		
	Expressed fear	.164**		
Perceived arousal	Proximal percepts (M3)		.905***	
	Loudness	.681***		
	Speech rate	.119***		
	Instability	.181***		
	Distal cues (AP)		.914***	.009***
	F0 floor	.069**		
	F0 range	.063*		
	Relative energy	-.078*		
	Expressed emotions (D)		.925***	.011***
	Expressed sadness	-.060*		
	Expressed anger	.105**		
	Expressed fear	.074*		
	Expressed arousal	.125***		

*Note*:

* = p < .02

** = p < .01

*** = p < .001.

Only p-values *<* .02 are reported.

Table 5 shows the results for the path groups M2 and EP (defined in Fig 4) with the proximal percepts as the dependent variable. The path group M2 allows assessment of the contributions of the distal cues with regard to the proximal percepts or, in terms of the TEEP model, the transmission process. For example, intonation is predicted by mean intensity (*b* = .305), intensity range (*b* = .127), fundamental frequency (F0 floor 5^th^ percentile; *b* = .176), and frequency range (F0 range; *b* = .288). The path group EP shows the importance of the expressed emotions for predicting a proximal percept in addition to the acoustic measures. Intonation, for example, is predicted by low anger (*b* = -.180), low sadness (*b* = -.252), low fear (*b* = -.187), and high arousal (*b* = .172). No strict one-to-one relationship between acoustic measures and proximal counterpart is detected except for perceived mean intensity—loudness and duration—speech rate.

**Table 5:** Prediction of *proximal percepts* by distal cues and expressed emotions, including standardized partial regression coefficients, *R* ^2^, and incremental *R* ^2^.

Proximal percept (DV)	Significant predictors (*p* = < .02)	Standardized partial regression coefficient *b*	*R* ^2^	Δ*R* ^2^
Intonation	Distal cue (M2)		.630***	
	Intensity mean	.305***		
	Intensity range	.127**		
	F0 floor	.176***		
	F0 range	.288***		
	Expressed emotion (EP)		.684***	.054***
	Anger	-.180***		
	Sadness	-.252***		
	Fear	-.187***		
	Arousal	.172**		
Loudness	Distal cue (M2)		.916***	.
	Intensity mean	.752***		
	Intensity range	.095***		
	Relative energy	-.082**		
	Expressed emotion (EP)		.919***	.003*
	Expressed anger	.077**		
Pitch	Distal cues (M2)		.645***	
	Intensity mean	.388***		
	F0 floor	.374***		
	F0 range	.322***		
	Expressed emotion (EP)		.697***	.052***
	Anger	-.325***		
	Fear	-.096*		
Roughness	Distal cue (M2)		.078***	
	Intensity range	-.167*		
	Duration	.167*		
	Relative energy	-.243**		
	Expressed emotion (EP)		.160***	.082***
	Anger	.346***		
	Sadness	.224**		
	Fear	.171**		
	Arousal	.295**		
Speech rate	Distal cues (M2)		.644***	
	F0 range	.104*		
	Duration	-.542***		
	Expressed emotion (EP)		.711***	.067***
	Fear	.289***		
Instability	Distal cues (M2)		.314***	
	F0 floor	.174**		
	F0 range	.181***		
	Duration	.223***		
	Expressed emotion (EP)		.616***	.302***
	Anger	-.421***		
	Sadness	.274***		
	Fear	.184***		
	Arousal	.214**		

*Note*:

* = p < .02

** = p < .01

*** = p < .001.

Only *p*-values *<* .02 are reported.

Table 6 shows the relationship between the expressed emotions and the acoustic measures as the dependent variable. The path group M1 describes the externalization process in terms of the TEEP model. For example, mean intensity (loudness) is positively associated with anger (*b* = .340), negatively associated with sadness (*b* = -.135), and highly positively associated with arousal (*b* = .766).

**Table 6:** Prediction of *distal cues* by expressed emotions including standardized partial regression coefficients and *R* ^2^.

Distal cue (DV)	Significant predictors (*p* = < .02)	Standardized partial regression coefficient *b*	*R* ^2^
Intensity mean	Expressed emotion (M1)		.748***
	Anger	.340***	
	Sadness	-.135***	
	Arousal	.766***	
Intensity range	Expressed emotion (M1)		.321***
	Anger	.160**	
	Arousal	.519***	
F0 floor	Expressed emotion (M1)		.508***
	Fear	.195***	
	Arousal	.684***	
F0 range	Expressed emotion (M1)		.298***
	Anger	.148**	
	Fear	-.148**	
	Arousal	.477***	
Duration	Expressed emotion (M1)		.068***
	Anger	-.176**	
	Fear	-.283***	
Relative energy	Expressed emotion (M1)		.516***
	Anger	-.317***	
	Sadness	.108**	
	Arousal	-.619***	

*Note*:

* = p < .02

** = p < .01

*** = p < .001.

Only p-values *<* .*02* are reported.

In Tables 4 to 6, we computed for each dependent variable the amount of variance that is explained by the respective set of predictors in a stepwise regression. For example, Table 4 indicates that the amount of variance in perceived anger that is explained by the proximal percepts is *R* ^2^ = .638. If the distal cues are added, this amount increases to *R* ^2^ = .655. Adding the expressed emotions increases the *R* ^2^ to .762. Although anger and arousal (*R* ^2^ = .905) are relatively well explained by the model, this is only moderately the case for happiness (*R* ^2^ = .265). For the proximal percepts, Table 5 shows that roughness is accounted for only marginally by the predictors (*R* ^2^ = .160), and for the acoustic measures, Table 6 indicates that mean intensity (acoustic loudness) is well explained by the expressed emotions (*R* ^2^ = .748), whereas this is not the case for acoustic duration (*R* ^2^ = .068).

The incremental *R* ^2^ values in Table 4 are especially interesting in judging the importance of the distal cues and the expressed emotions once the proximal percepts are taken into account to explain the perceived emotions. As Table 4 shows, adding the distal cues does not improve the prediction of the perceived emotions and arousal substantially.

**Fig 5:**
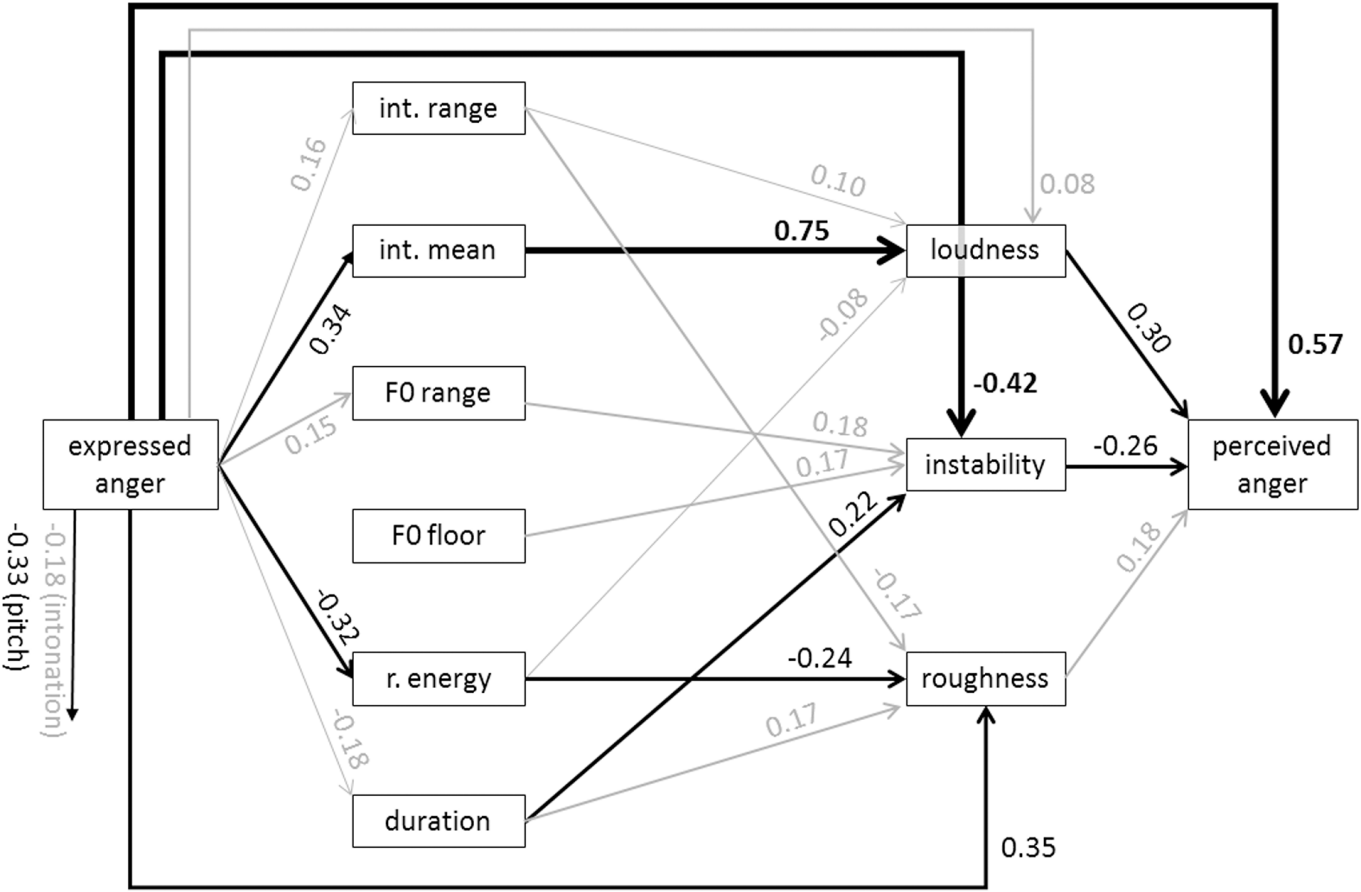
Standardized path coefficients of the estimated model for anger (data merged for MUC and GVA). Only significant path coefficients are shown (*p* < .02). Significant paths with an absolute value > .2 are depicted in black, significant paths with an absolute value < .2 are depicted in black. int. = intensity; r. energy = relative energy.

**Fig 6:**
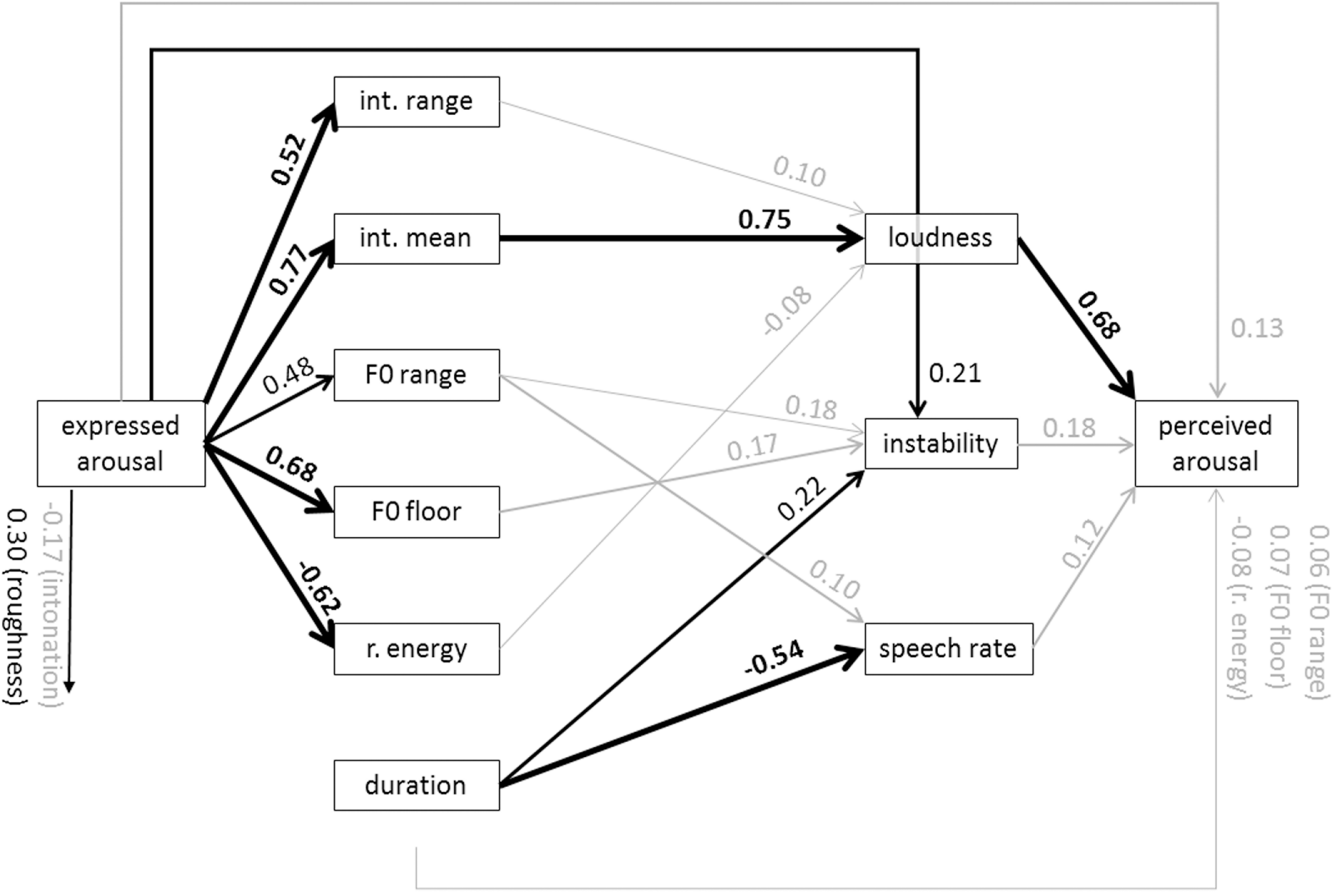
Standardized path coefficients of the estimated model for arousal (data merged for MUC and GVA). Only significant path coefficients are shown (*p* < .02). Significant paths with an absolute value > .2 are depicted in black. Significant paths with an absolute value < .2 are depicted in black. int. = intensity; r. energy = relative energy.

**Fig 7:**
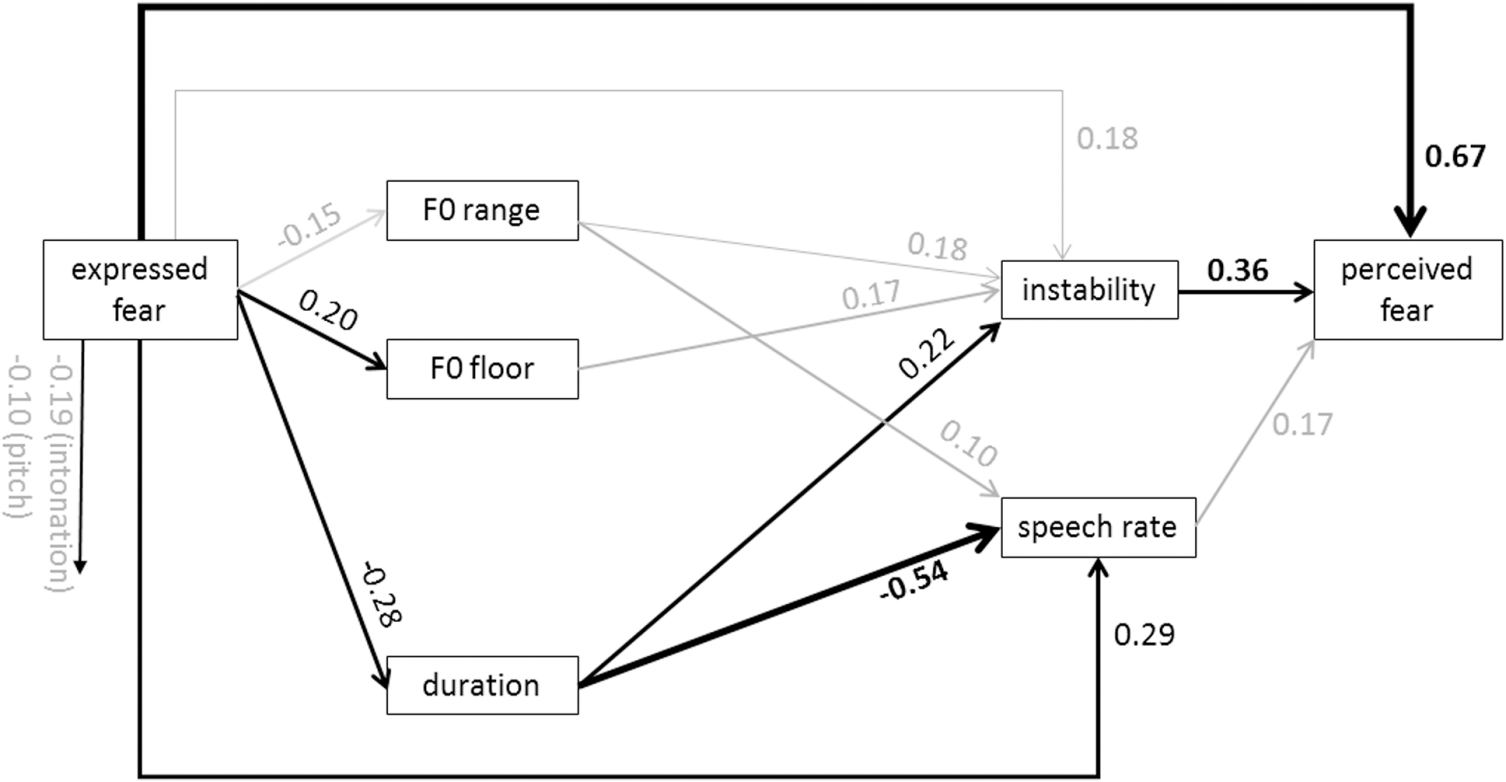
Standardized path coefficients of the estimated model for fear (data merged for MUC and GVA). Only significant path coefficients are shown (*p* < .02). Significant paths with an absolute value > .2 are depicted in black. Significant paths with an absolute value < .2 are depicted in black.

**Fig 8:**
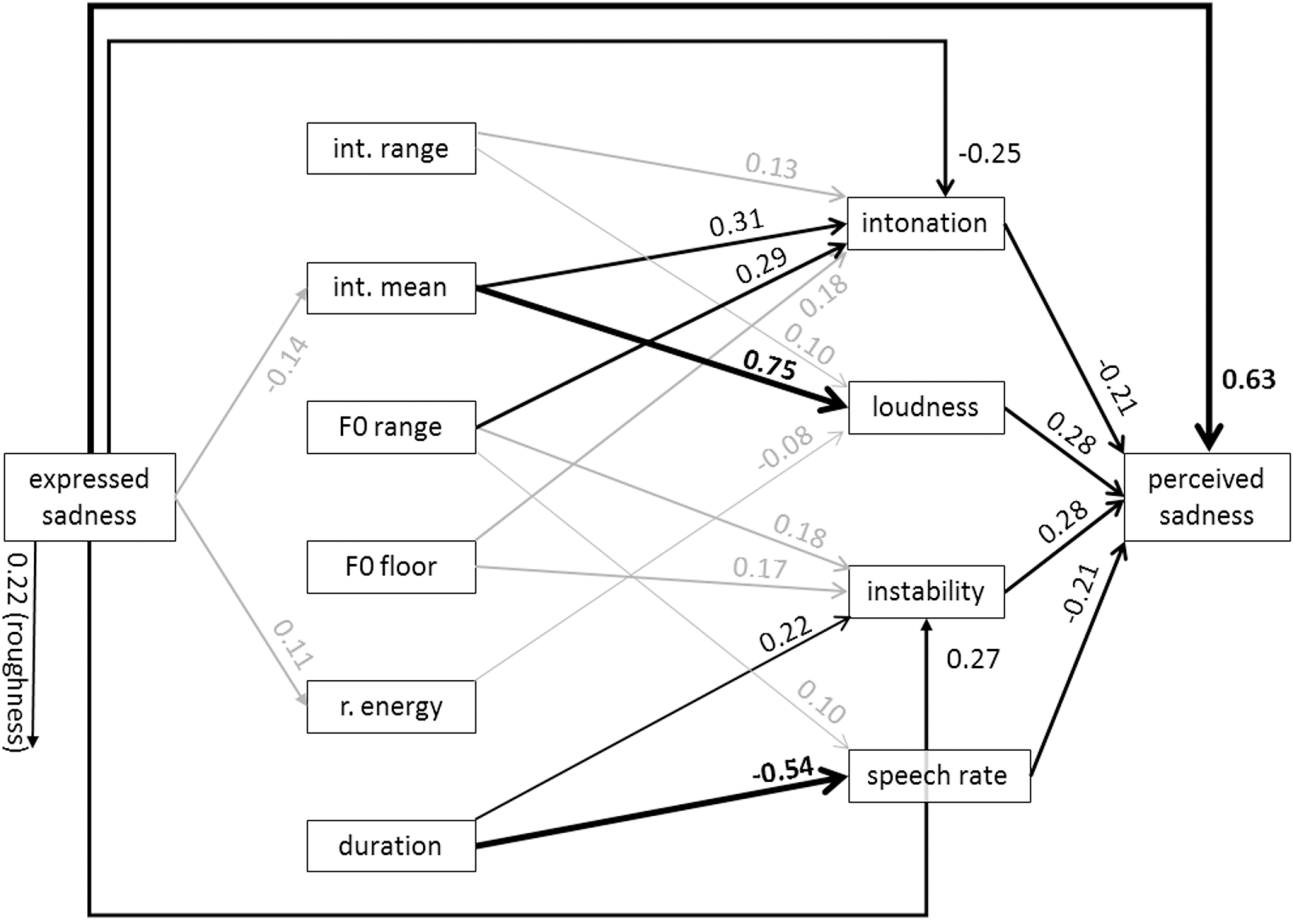
Standardized path coefficients of the estimated model for sadness (data merged for MUC and GVA). Only significant path coefficients are shown (*p* < .02). Significant paths with an absolute value > .2 are depicted in black. Significant paths with an absolute value < .2 are depicted in black.

Figs 5 and 6 show the specific models for anger and arousal. From the graph for anger, it is evident that the most dominant path chain from expressed anger to perceived anger runs from high acoustic intensity to high perceived loudness and from there to the inference of perceived anger. However, the direct path from expressed anger to perceived anger is relatively strong, indicating that the acoustic measures and the proximal percepts do not carry all the information that is used to infer the emotion. Fig 6 for arousal shows that high arousal is reflected in specific changes in almost all acoustic measures except for relative energy and duration. On the proximal side of the model, it is mostly loudness that is used to infer perceived arousal. Figs 7 and 8 show the results for fear and sadness. Fig 7 shows that fear portrayals differ from happiness portrayals by a lower F0 range, a higher F0 floor and lower duration. Expressed fear is negatively associated with duration, suggesting higher tempo. Correspondingly, duration is negatively associated with perceived speech rate and positively with perceived instability. Finally, high perceived instability and high perceived speech rate are associated with perceived fear. Fig 8 shows that the acoustic measures included in the model are only weakly associated with expressed sadness. The strongest paths between the acoustic measures and proximal percepts run from mean intensity to intonation and loudness. Perceived sadness is negatively associated with intonation modulation and speech rate, and positively with perceived instability.

Table 7 shows the direct, total indirect, and total effects for the emotion families (the effects are estimated with Mplus using robust standard errors, which are shown in parentheses in Table 7; total effect = total indirect effects + direct effect). The total indirect effects reflect mediation via the acoustic measures and the proximal percepts to detect an emotion. These coefficients are relatively low except for arousal. In addition, the relatively high direct effects indicate that the communication process is not sufficiently captured by the variables used. Mediation for happiness could not be assessed, as happiness was used as a reference category. An analysis for happiness (contrasted with all other emotions) is provided in the Supplementary Information (Tables D and E in S2 File—Data).

**Table 7:** Standardized direct, total indirect, and total effects from expressed emotion to perceived emotion.

Effects	Anger	Fear	Sad	Arousal
Total indirect effects	.274*** (0.038)	.161*** (0.030)	.115*** (0.029)	.664*** (0.031)
Direct effect	.566*** (0.050)	.668*** (0.046)	.630*** (0.049)	.125*** (0.029)
Total effect	.840*** (0.030)	.829*** (0.034)	.746*** (0.038)	.789*** (0.022)
Total indirect effects/Total effect	.326	.194	.183	.842

*Note*:

*** = p < .001.Standard errors in brackets.

## Discussion

Overall, the results show that (a) the selected vocal parameters (distal/acoustic and proximal/perceived) are differentially related to different emotions and (b) they allow a more comprehensive account of the communication of the arousal dimension than of the differential quality of the emotion families (with the exception of anger). In what follows, we briefly discuss the main findings, first for the LME and then for the path analysis.

### LME Modeling

The results obtained for the analyses with the classical LME indicate that the vocal communication of emotion can be largely accounted for within the framework of the Brunswikian lens model. In both data sets, the receiver’s emotional attributions could be partly accounted for either by the objectively measured acoustic parameters, or by the proximal percepts (as indicated by large *R*_*s*_ coefficients). Likewise, the sender’s expressed emotion could be partly accounted for by the acoustic parameters or by the proximal percepts (as indicated by large *R*_*e*_ coefficients). The large coefficients of communication achievement (*r*_*a*_) indicate that the intended emotional expressions of the speakers were to a large extent recognized by the decoders. The matching coefficients (*G*) were overall also very large, indicating that the use of the voice cues in emotion externalization (expression) is symmetric to the use of the voice cues on the receiver side (for emotional attributions). This observation supports hypotheses postulating symmetrical processes in expressive behavior on the one hand and in perception on the other. High coefficients for speaker and rater consistency (*R*_*e*_, *R*_*s*_) show that the externalized emotions and the participant’s emotional attributions are relatively strongly related to the eight acoustic parameters or the perceived voice cues, respectively.

In both data sets, the perceived voice cues appeared to be better predictors for the expressed emotions and the emotional attributions than the distal acoustic parameters. This observation is consistent with the results of van Bezooijen [49], who reported that ratings of vocal cues could better discriminate emotional expressions than could ratings of acoustic cues. This observation is interesting, given that subjective ratings are a priori less reliable measures (due to inter-individual differences, biases in ratings, and intrapersonal inconsistency issues) than objectively measured acoustic parameters.

### Path Analysis Based on the TEEP Model

One of the central questions in this research concerns the degree to which the voice cues (distal or proximal) can account for the variance in perceived emotions. The results (see Table 5) show that overall, the *R* ^2^ are large: For all emotion families, about 60% to 70% of the variance (more than 90% in the case of arousal) can be explained by the complete predictor set, which includes also expressed emotions and expressed arousal. In contrast, there are marked differences between emotions in the degree to which the mediating predictors, distal and proximal variables, account for the explanatory power. The results for the hierarchical regression analysis allow partitioning of the total *R* ^2^ into the relative contribution of the respective predictor sets (see Table 7). The proximal percepts were entered into the hierarchical regressions first, accounting for 91% of the variance for arousal and 64% for anger, followed by 49% for fear and 41% for sadness and 27% only for happiness.

Compared with the LME approach (where separate analyses had to be computed for proximal and distal variables), the TEEP model allows a more integrative approach. The results generally show that, for all emotions, the distal variables do not explain much of the variance once the proximal variables are entered into the model. This corresponds to theoretical expectations, as one can assume that the proximal cues, the voice percepts, are in fact directly based on the acoustic information carried by the wave form. Most of the valid information provided by the acoustic cues ought to be available in the proximal percepts, provided that they reflect the same vocal features. Tables 4 to 6 show the details of these relationships. The results show three important patterns:

1. There are only a few one-to-one relationships (i.e. a particular acoustic parameter exclusively corresponding to a parallel dimension in voice perception). With the exception of acoustic intensity or energy (intensity mean) accounting for most of the loudness judgments, in all other cases, the subjective voice perception dimensions are determined by several acoustic parameters, suggesting that the perceptual dimensions important for emotion recognition in the voice are defined by interactions between different acoustic cues.
2. The combination of acoustic parameters that best predicts a proximal percept varies over different proximal scales.
3. Except in the case of loudness, the distal cues account only for 60% or less of the variance in the proximal percepts (only about 30% for instability and a very low 8% for roughness). This means that additional acoustic parameters need to be identified and measured in order to understand how exactly distal acoustic cues determine the subjective impression of voice and speech quality. This is particularly true for voice quality dimensions such as instability and roughness that are not easily characterized on the acoustic level in running speech. Most likely, the subjective judgments on these scales depend on complex combinations of acoustic cues, including parameters that have not been included in the current analysis such as perturbation measures, indices of glottal functioning [11], or articulatory phenomena like mouth shape variations that change radiation. Part of the variance not explained by the measured acoustic cues carries important information concerning the type of expressed emotion, as shown by the fact that additional variance in the proximal percepts is explained by direct paths from one or more expressed emotions after the effect of the acoustic cues has been partialed out (see Table 5).

The graphic representation of the results for negative emotions and arousal in Figs 5–8 demonstrates the advantages of the TEEP model as outlined in the introduction. It charts the complete process of expression, transmission, and inference and allows one to determine where the model supports earlier predictions and where further improvements need to be made.

For anger (Fig 5), the strongest complete mediation path (M1+M2+M3 in Fig 4) is found for the expression of anger through high acoustic intensity, leading to the impression of a loud voice giving rise to the inference of anger. A second such path is constituted by flattening of the spectral slope (i.e. a decrease in relative energy in the frequencies below 1000 Hz with a corresponding increase of the higher frequencies), which might, together with other changes, lead to an increase in the perception of “roughness” of the voice and then also be interpreted as anger. Both of these paths have been theoretically postulated. Thus, Scherer (see Table 6 in [50]) presented a complete set of theory-based acoustic predictions for major emotions, in which an increase in intensity and high frequency energy is predicted for anger (although the mediation through proximal percepts was not yet specified). An additional prediction suggested an increase in F0 range and variability. This is also confirmed by the present data for the distal path, anger expression leading to an increase in the range of F0. All of these predictions have also been empirically confirmed in the literature (see for example the review by Juslin & Laukka, [3]; see also Table A in S1 File—Appendix). In the present case, these acoustic parameters may contribute to the proximal impression of instability in the voice, which is interpreted as a counter indication of the presence of anger (negative path coefficient).

This apparently discrepant result demonstrates another advantage of the path analytic TEEP model—the generation of new research questions. As noted earlier, the instability dimension is currently not well explained by acoustic parameters and it is thus difficult to interpret the reason for the apparent discrepancy. Most likely, there is an interaction between F0 range on the one hand and F0 floor and acoustic duration on the other, as all three parameters show a positive effect on instability (i.e. high F0 floor and high variability with a slow speech rate are seen as instable). However, anger produces faster speech (as also predicted by Scherer [50], and empirically confirmed in earlier studies [3]). It is difficult to interpret these inconsistencies given our current knowledge. Further research is required to disentangle the sources of vocal instability perception and to identify further parameters, including perturbation measures such as jitter, shimmer, or the harmonic-to-noise ratio. The strongly negative semi-direct path EP, bypassing the distal level, from expressed anger to instability, shows that an angry voice is not perceived as instable, which is consistent with the negative effect of instability on anger perception. In any case, these inconsistencies may have reduced the amount of variance explained by the complete indirect mediation paths of type M1+M2+M3, thus accounting for the relatively strong direct path D. On the whole, however, the model is successful for anger and provides strong support for both the feasibility of the modeling of the inference process by the TEEP model and for the earlier predictions based on the component process model of emotion (as described in [50]).

The model provides an excellent account for the communication of emotional arousal (as shown in Fig 6). Here both the direct (D) and semi-direct paths (EP and AP) are of little import, with over 90% of variance being explained by the indirect mediation paths (M1+M2+M3), essentially through the loudness and instability percepts and due to the strong effects of expressed arousal on the underlying acoustic parameters F0 and intensity, both with respect to mean and range. This underlines the important role of strong distal connections between different emotions and specific configurations of acoustic parameters. The indirect mediation paths also provide some indication that, in this case, there is a more coherent meaning for the instability dimension, a consistent clustering of F0 floor and range as well as duration (slow speech rate), even though there is no relationship of the latter with expressed arousal.

These results reflect the strong evidence from past work in this area suggesting that the voice is the privileged modality for the expression and communication of arousal and activation, whereas the face is vastly superior with respect to valence [5]. It seems plausible that studies involving “extreme” emotional variation (emotion portrayals are often exemplars of very strong and prototypical emotions) will always find emotional arousal to be a “higher order factor” in the voice, given that more emotional arousal is likely to be translated in increased vocal effort and faster speech, which in turn affects many vocal cues. The lack of clearly identified valence cues is also brought out clearly by the current results, the modeling of the happiness family being by far the least satisfactory. This is despite the fact that the accuracy scores for happiness are not much below the average of the other emotions. To the extent that results cannot be explained by guessing strategies given the small number of positive emotions, listeners seem to have been able to correctly infer happiness from voice and speech in our corpora (suggesting that relevant cues were available to them). However, so far we have little evidence concerning the distal and proximal cues that the inference process is based on. One possibility might be the change in lip radiation patterns while speaking with a smile [51], or the size-code hypothesis [52] which implies that higher sociability (and positive emotionality) may be indicated by higher F0 level and larger formant spacing consecutive to shortening of the vocal tract [53].

The results for sadness and fear are somewhat better than for happiness, but in each case, the variance explained by the indirect paths explains less than 50% of the variance in the perceived emotions. The differentiation is provided by acoustic duration/speech rate, decreasing for sadness and increasing for fear (as reported in earlier studies [3]). In both cases, perceived instability is involved, with an increase making both fear and sadness judgments more likely. This, and the negative relationship for anger, might suggest that perceived instability is seen as an indicator of low power or helplessness. It seems promising to examine this dimension of voice perception more closely.

### Limitations

The expression corpora and corresponding data sets used for the analyses reported here are the first to allow integration of the four sets of measures (expressed emotions, distal cues, proximal cues, and perceived emotions) into single models of vocal emotion communication. However, the nature of the two data sets used here also imposes limitations on our analyses. Sources of limitations, imposed by the established design of emotion recognition studies, include the use of binary variables (present/absent) for the operationalization of expressed emotions, the absence of neutral comparison stimuli (all portrayals represent emotional expressions), and the sample sizes, which are small in relation to the large number of potentially relevant cues.

The ratings of voice cues and ratings of emotions have been obtained from different raters, using a design that a priori precludes the possibility that the emotional ratings might have conditioned the ratings of voice cues. However, it cannot be excluded that the participants who rated the voice cues were influenced by spontaneously occurring implicit emotion judgments.

Furthermore the use of independent ratings (for voice cues and perceived emotions) does not allow modeling the perception process with respect to individual listeners (and variance across listeners). In the current models the variance was considered only across emotion portrayals, individual perceptual processes are not addressed.

The optimal choice of vocal cues (usually acoustic summaries of the speech signal) is a recurrent problem in this research domain. There is an urgent need to develop a principled selection method to allow testing of specific hypotheses. In particular, it seems that different sets of cues might be distinctive for different emotions and that more work is needed to identify the vocal cues that are best suited to describe various emotions in speech. The choice of acoustic measures included in the models described above was limited, lacking, for example, measures relative to formants or voicing stability. Some frequently used spectral measures were included, but there is certainly room for refinement. In general, the field needs a better mapping of the pertinent vocal features involved in the communication of emotion (see Eyben et al. [54] for a concerted action of the voice research community in this respect).

Furthermore, the acoustic variables included in our analyses have not been selected (or transformed) for perceptual relevance or saliency. For example, the variations induced by emotion in some of the parameters might fall below discrimination thresholds. Further research should consider adding a psychoacoustic level of representation to the model, situated between the distal and the proximal cues. This could consist of appropriate transformations of the acoustic variables in order to make them more perceptually relevant (e.g. represent F0 in semi-tones or integrate spectral measure with amplitude measures in order to better approximate perceived loudness).

Finally, in recent years the use of emotion portrayals has become a common concern. However, the use of *enacted* material, in an attempt to get as close as possible to natural expressions, is obligatory in this research domain, as it is practically and ethically impossible to obtain a large set of vocalizations for 12 to 18 emotions from the same person in real life. Using a convenience sample of vocal emotion expressions from different individuals recorded on the fly is ruled out by the fact that individual voice qualities are extremely variable, making it impossible to compare acoustic emotion variations across speakers.

### Conclusion

The promise of using mediational analysis (in particular path analysis) to understand the processes of expression and impression in social perception—in contrast to designs focusing only on expression or encoding or only on inference and decoding—is increasingly recognized. Most importantly, such research designs focus on the identification of the cues that carry the pertinent information. This approach is of particular importance in the area of vocal markers of identity, personality and emotion given the wealth of information provided by the voice and the complexity of the acoustic markers [6]. A pertinent example is a recent study showing that women listeners use sexually dimorphic voice cues which correlate with speakers' variation in stature and hormonal status to judge the masculinity of male speakers, highlighting the interdependence of physiological, acoustic and perceptual dimensions [55].

In a similar vein, the present paper shows the utility of applying a modified Brunswikian lens model (TEEP) that separately measures both distal and proximal voice cues to assess the transmission and recoding process, to the vocal communication of emotion. We reported secondary analyses of two data sets that include proximal/perceived voice cues and distal/acoustic measures obtained for two corpora of vocal emotion portrayals. Our main goal was to highlight how the communication process and the contribution of various cues (distal or proximal) can be represented by using LMEs and path analyses.

The statistical models (LME and path analysis) presented in this paper indicate that this approach to the study of vocal communication of emotion is highly feasible and that the distal and proximal variables used in the models mediate the communication of arousal and negative emotions to a large extent. From the set of proximal percepts, only intonation modulation was indicative for perceived happiness, reflecting the frequently observed absence of specific valence cues in the voice. However, recognition accuracy was comparably large for all emotions, as reflected by strong direct paths from expressed to perceived emotion.

The expected arousal dominance showed up clearly in our models (in the LME and the path analysis). However, the current results contribute to our understanding of the underlying mechanisms. Emotions characterized by high arousal are characterized by high intensity and high F0 floor and range on the distal side, with corresponding perceptions of loudness and high pitch. This evidence for highly efficient information transmission suggests the existence of inference rules that directly correspond to the empirical associations we found. In contrast, high speech rate, often predicted as a marker of arousal, does not consistently produce the same pattern.

Importantly, the current work has increased our understanding of the cues that are specific to *emotion families*, independent of potential arousal differences among the family members (e.g. irritation, cold anger vs. rage, hot anger). We suggest that the cues specific to the anger family as a whole, irrespective of arousal differences, are the following: a comparatively high intensity, a flat spectral slope (perceived as roughness), and a firm, steady voice (the acoustic correlates of which remain unclear for the moment, although perturbation is a likely candidate).

In sum, we have shown that accounting for the process of vocal emotion communication using the Brunswik-based TEEP model is a promising approach to understanding the processes of emotion production and recognition. It provides insight into which specific cues are used to express an emotion, how the distal indicator variables map to proximal percepts, and which proximal percepts are used to recognize a certain emotion. In particular, our results encourage the search for hitherto unexploited distal voice cues associated with emotions, carrying essential discrimination information for proximal percepts (e.g. instability). A first step in this direction is currently undertaken in the form of the specification of a standardized set of acoustic parameters for research on vocal expression of emotion [54]. This is particularly pertinent in the case of positive emotions, which have been repeatedly shown to be difficult to characterize by acoustic parameters despite the fact that raters can identify them rather accurately. Distal acoustic cues with a better match to speech production and/or speech perception mechanisms will be needed to improve our models. Correspondingly, further studies of perceived voice cues need to be conducted to be able to include not only a complete set of distal, but also appropriate proximal measures in such models. The TEEP model also suggests new possibilities for research designs using increasingly sophisticated technological tools for voice manipulation through synthesis and morphing, as based on emotion portrayals or realistic speech data. Although this article has focused on vocal communication, this framework can be extended to other areas of interpersonal communication such as the nonverbal communication of emotions in facial or gestural expressions.

The study of the perception and inference of emotion from nonverbal expressions continues to be highly popular in psychological emotion and social perception research. Unfortunately, much of this research is narrowly confined to the study of recognition accuracy, with little concern for the underlying mechanisms and processes or the nature of the acoustic cues and their perception by listeners. The widening gulf between production studies (which are relatively rare) and recognition studies (which exist in abundance) constitutes a major limitation for progress in this field, particularly with respect to understanding the underlying communication process. Arguably, this research could greatly benefit by a comprehensive, process-oriented approach informed by a theoretical framework and incorporating production and transmission as well as perception and inference. Similarly, the recent surge of activity in the domain of *affective computing* (involving both engineering and computer sciences), could benefit from the type of modeling described here. This is particularly true for machine learning approaches to automatic emotion detection in the voice and for realistic vocal emotion expression synthesis for avatars in the context of robotics or human-machine-interaction. It is to be hoped that the demonstration of the utility and feasibility of a comprehensive path modeling approach motivates researchers from a wider area in perception and cognition to turn to this important aspect of emotion communication in human social interaction.

## Supporting Information

S1 File Appendix. Details on methods and procedures.
(PDF)

S2 File Data. Comprehensive listing of statistical results.
(PDF)

S3 File Raw data file. Raw data used for the path analysis.
(TXT)
