# Supplementary material for: Path Models of Vocal Emotion Communication
Source: PLoS One. 2015 Sep 1;10(9):e0136675. doi: 10.1371/journal.pone.0136675 (PMC4556609; doi:10.1371/journal.pone.0136675)
Supplement: S1 File — Details on methods and procedures. (PDF) [file pone.0136675.s001.pdf]

Supplemental information for

Baenziger et al. - *Path Models of Vocal Emotion Communication* - PlosOne

S1 – Appendix

Table A. Summary of results reviewed by Juslin and Laukka (2003, pages 792-799).

Parameter	Anger	Fear	Happiness	Sadness	Tenderness
intensity (mean)<br>(high - med - low)	high<br>(30/32)	high<br>(11/22)	high<br>(20/26)	low<br>(29/32)	low<br>(4/4)
intensity variability<br>(high - med - low)	high<br>(9/12)	high<br>(7/12)	high<br>(8/13)	low<br>(8/11)	
F0 (mean)<br>(high - med - low)	high<br>(33/43)	high<br>(28/39)	high<br>(34/38)	low<br>(40/45)	low<br>(4/5)
F0 variability<br>(high - med - low)	high<br>(27/35)	low<br>(17/32)	high<br>(33/36)	low<br>(31/34)	low<br>(5/5)
F0 contours<br>(raising - falling)	raising<br>(6/8)	raising<br>(6/6)	raising<br>(7/7)	falling<br>(11/11)	falling<br>(3/4)
high-freq. energy<br>(high - med - low)	high<br>(22/22)	high<br>(8/16)	high<br>(13/17)	low<br>(19/19)	low<br>(3/3)
speech rate<br>(fast - med - slow)	fast<br>(28/35)	fast<br>(24/29)	fast<br>(22/33)	slow<br>(30/36)	slow<br>(3/4)
microstructural regul.[a]<br>(regular - irregular)	irregular<br>(3/3)	irregular<br>(2/2)	regular<br>(2/2)	irregular<br>(4/4)	regular<br>(1/1)
proportion of pauses<br>(large - med - small)	small<br>(8/8)	small<br>(4/9)	small<br>(3/6)	large<br>(11/12)	large<br>(1/1)
precision articulation<br>(high - med - low)	high<br>(7/7)	?[b]<br>(6)	high<br>(3/5)	low<br>(6/6)	low<br>(1/1)
formant 1 (mean)<br>(high - med - low)	high<br>(6/6)	low<br>(3/4)	high<br>(5/6)	low<br>(5/6)	
formant 1 (bandwidth)<br>(narrow - wide)	narrow<br>(4/4)	wide<br>(2/2)	narrow<br>(2/3)	wide<br>(3/3)	
jitter<br>(high - low)	high<br>(6/7)	?[b]<br>(8)	high<br>(5/8)	low<br>(5/6)	
glottal waveform<br>(steep - rounded)	steep<br>(6/6)	rounded<br>(4/6)	steep<br>(2/2)	rounded<br>(4/4)	

The proportion of studies reporting the result is indicated in each cell in parentheses.

[a] Microstructural irregularities are defined as short-term irregularities of F0, intensity, and/or duration. Irregularities are theoretically associated with negative emotions.
[b] Equal numbers of studies reported different levels for this emotion and this vocal description (the number of studies is shown in parentheses).

Juslin PN, Laukka P. Communication of emotions in vocal expression and music performance: different channels, same code? Psychol Bull. 2003;129: 770–814.

Methods

(A) Extraction of acoustic parameters

Description adapted from

Bänziger T, Patel S, Scherer KR. The role of perceived voice and speech characteristics in vocal emotion communication. J Nonverbal Behav. 2014;38: 31–52.

All selected portrayals for the GVA and the MUC corpora were acoustically analyzed using PRAAT (open access software developed by Boersma and Weenink) to extract a set of standard acoustic parameters. The scripts used for the acoustic analyses can be downloaded at the following address: http://www.affective-sciences.org/gemep/perceived_voice. Further supplementary materials (audio examples and details of statistical results) are also available at the same address.

MUC corpus. Fundamental frequency (F0) was extracted using PRAAT's auto-correlation algorithm. A "conservative" manual correction of the F0 contour was performed. Detection errors were corrected where the algorithm detected periodicity in unvoiced parts of the signals. The recordings were manually segmented so as to identify pauses (speech interruptions), as well as voiced and unvoiced segments. Several parameters were extracted from the F0 and several absolute and relative duration parameters were computed for different speech segments and pauses. Further parameters were extracted from the intensity contour. The proportion of spectral energy in various frequency regions of the long term averaged spectrum (LTAS) was also investigated. The spectrum was segmented into bands, matching the approach and results reported by Banse and Scherer (1996). Spectral parameters were extracted separately for the voiced and the unvoiced parts of the expressions. In total, 44 parameters were extracted from the signals, many of which were very highly intercorrelated. All parameters were independently standardized within speaker in order to control for variations due to inter-individual differences (using z-transformations).

GVA corpus. The parameters described for the MUC coprus were extracted for the vocal portrayals used in Study 2. However, no manual corrections of F0 or duration were performed in

Study 2, as the effects of the manual corrections in Study 1 were estimated to be negligible. Several measures on the long term average spectrum (LTAS) included in Study 1 (various spectral bands on the voiced and the unvoiced spectrum) were not used in the analyses, since they did not make significant independent contributions to the differentiation of emotions in Study 1. In addition it appeared that some additional parameters could be reliably extracted. Shimmer, jitter, and harmonics-to noise ratio (HNR) were extracted and added to the parameter set. All parameters were standardized separately within speakers (using z-transformations).

(B) Selection of acoustic parameters.

Acoustic parameters are selected based on the results of a Principal Component Analysis, 44 extracted acoustic parameters are used as the input of the analysis (acoustic measures extracted for the MUC-corpus).

Table B. List of acoustic parameters originally extracted for the MUC corpus.

Code	Description	Code	Description
int.min	intensity min., measured in dB	For voiced segments	
int.max	intensity max., measured in dB	v.125-200	% spectral energy between 125 et 200 Hz
int.range	(int.max-int.min), intensity range (dB)	v.200-300	% spectral energy between 200 et 300 Hz
int.moy	intensity mean, measured in dB	v.300-500	% spectral energy between 300 et 500 Hz
int.sd	standard deviation of intensity (dB)	v.500-600	% spectral energy between 500 et 600 Hz
		v.600-800	% spectral energy between 600 et 800 Hz
F0.min	minimum F0 (Hz)	v.800-1k	% spectral energy between 800 et 1000 Hz
F0.max	maximum F0 (Hz)	v.1k-1.6k	% spectral energy between 1000 et 1600 Hz
F0.range	(F0_max-F0_min), F0 range (Hz)	v.1.6k-5k	% spectral energy between 1600 et 5000 Hz
F0.moy	F0 mean (Hz)	v.5k-8k	% spectral energy between 5000 et 8000 Hz
F0.sd	standard deviation F0 (Hz)	v.0-500	% spectral energy below 500 Hz
F0.05c	5th centile F0 (Hz)	v.0-1k	% spectral energy below 1000 Hz
F0.25c	25th centile F0 (Hz)	Hamm	Hammarberg index, intensity max. between 0 et 2 kHz minus intensity max. between 2 et 5 kHz
F0.50c	median F0 (Hz)		
F0.75c	75th centile F0 (Hz)		
F0.95c	95th centile F0 (Hz)	For unvoiced segments	
F0.05-95c	(F0_95c-F0_05c), F0 range (Hz)	n.125-250	% spectral energy between 125 et 250 Hz

		n.250-400	% spectral energy between 250 et 400 Hz
dur.s/tot	duration of silent segments on total duration	n.400-500	% spectral energy between 400 et 500 Hz
dur.n/tot	duration of unvoiced segments on total duration	n.500-1k	% spectral energy between 500 et 1000 Hz
dur.v/tot	duration of voiced segments on total duration	n.1k-1.6k	% spectral energy between 1000 et 1600 Hz
dur.v/art	duration of voiced segments on non-silent	n.1.6k-2.5k	% spectral energy between 1600 et 2500 Hz
dur.tot	total duration (in secondes)	n.2.5k-4k	% spectral energy between 2500 et 4000 Hz
		n.4k-5k	% spectral energy between 4000 et 5000 Hz
		n.5k-8k	% spectral energy between 5000 et 8000 Hz
		n.0-500	% energy below 500 Hz
		n.0-1k	% energy below 1000 Hz

Results of the analysis (PCA for the selection of acoustic parameters):

Table C. Eigenvalues of the components.

Compo nents	Before rotation			Varimax Rotation		
	eigenvalue	% variance	% cumulated variance	eigenvalue	% variance	% cumulated variance
1	15.73	35.75	35.75	7.78	17.68	17.68
2	4.64	10.54	46.29	4.62	10.51	28.19
3	3.04	6.90	53.19	4.54	10.32	38.51
4	2.83	6.44	59.62	4.10	9.31	47.82
5	2.53	5.76	65.38	3.94	8.96	56.78
6	2.06	4.67	70.05	3.59	8.16	64.94
7	1.78	4.05	74.10	2.49	5.66	70.60
8	1.55	3.52	77.62	2.22	5.05	75.66
9	1.20	2.73	80.35	2.07	4.69	80.35

Table D. Factor loadings (variables that were selected for further analyses are indicated in bold characters).

	Components								
Parameters	**1**	**2**	**3**	**4**	**5**	**6**	**7**	**8**	**9**
F0.sd	0.970								
F0.05-95c	0.966								
**F0.range**	**0.944**								
F0.95c	0.856	0.400							
F0.max	0.849	0.397							
F0.75c	0.753	0.501							
F0.moy	0.674	0.616							
F0.50c	0.653	0.592	0.313						
**F0.min**		**0.911**							
F0.05c		0.876							
F0.25c	0.460	0.750							
v.1.6k-5k			0.860						
**v.0-1k**			**-0.839**						
Hamm	-0.316		-0.748						
v.1k-1.6k			0.621					0.335	
v.5k-8k		0.316	0.523					-0.469	
v.800-1k			0.423						
**dur.tot**				**-0.844**					
dur.s/tot				-0.823					
dur.v/tot				0.791			-0.324		
int.min	0.319			0.666					
**int.moy**	**0.479**	**0.335**	**0.397**	**0.548**					
**n.0-1k**					**0.908**				
n.0-500					0.764	-0.352			
n.5k-8k					-0.688		-0.473		

n.400-500					0.642				
n.4k-5k					-0.592				
n.500-1k	0.341			0.339	0.554				
n.125-250					0.551				
n.1k-1.6k	0.421				0.486				
n.250-400		0.364			0.462	0.333			
int.sd						0.852			
**int.range**						**0.847**			
v.125-200		-0.318				-0.531			
int.max	0.455	0.334	0.303	0.363		0.520			
dur.n/tot						-0.330	0.763		
n.2.5-4k					-0.307		0.699		
**dur.v/art**				0.532		0.345	**-0.635**		
n.1.6k-2.5k						0.347	0.611		
**v.600-800**								**0.785**	
v.200-300								-0.662	
**v.300-500**									**-0.867**
v.500-600									0.752
v.0-500			-0.378					-0.426	-0.672

(C) Voice cue ratings - rating procedures and reliability

Ratings of perceived voice cues (proximal cues)

Description adapted from

Bänziger T, Patel S, Scherer KR. The role of perceived voice and speech characteristics in vocal emotion communication. J Nonverbal Behav. 2014;38: 31–52.

MUC corpus. As the rating procedure used for the MUC corpus was more time-consuming than a conventional rating procedure, four groups of listeners were recruited to evaluate subsets of the 144 vocal expressions selected from this corpus. The groups were composed of 15 to 16 first-year students in psychology at the University of Geneva. All raters had normal hearing capacity and participated in the study against course credit. Students were randomly allocated to one of four groups. Group 1 consisted of 14 women and two men (average age = 21.3 years, *SD* = 4.3); group 2 consisted of 10 women and five men (average age = 21.7,*SD* = 4.3); group 3 consisted of 13 women and two men (average age = 20.2, *SD* = 1.7); group 4 consisted of 11 women and four men (average age = 21.4,*SD* = 6.1). The study took place in a small laboratory for psychological assessment at the University of Geneva.

GVA corpus. Based on the results obtained for the MUC corpus, a simplified rating procedure (see below) was used for the ratings collected on the GVA corpus, involving nineteen raters (10 women and 9 men with an average age of 22.4 years, *SD* = 2.2) who were asked to assess all 160 portrayals in one rating session. The raters received a financial compensation of 60 CHF for their contribution. All participants had normal hearing. The study took place in a small laboratory for psychological assessment at the University of Geneva.

The ratings for the MUC and the GVA corpora were collected several years apart, using different procedures to collect the ratings.

MUC corpus. It has been shown that the evaluation of vocal quality on scales such as "rough" or "breathy" do not yield reliable judgments (inter-rater reliability and test-retest reliability were found to be low). Internal standards of comparison (anchors) used by listeners when they are making judgments vary from one listener to another and also vary over time for a

single listener. In order to address the problem of variable anchors, we adapted a rating procedure introduced by Granqvist [Granqvist S. (1996). Enhancements to the visual analogue scale. Speech, Music and Hearing - Quarterly Progress and Status Report. 1996; 4: 61-65] , in which all expressions produced by one speaker can be rated simultaneously and direct comparison is used to ensure that the standard for comparison is not fluctuating for a given speaker. Using this approach, a visual analog scale was presented to the listeners on a computer screen. The task of the listeners was to place the vocal expressions on this scale. All expressions produced by a given speaker appeared on the screen in random order, as identical icons, which could be played by double-clicking on the icons. The raters' task was to place them on the scale depending on the value he or she allocated to each recording. Listeners were free to listen to the vocal expressions again as often as they wished, and could modify their answers. The vocal expressions produced by different speakers were presented on successive screens, so that the judgments were relative to the range of variation of a given speaker (and insensitive to inter-speaker differences). In addition, two recordings illustrating the ends of each scale were presented at the bottom of the screen. Those recordings were presented as illustrations and were not meant to be used as anchors. They can be accessed along supplementary materials on the website indicated above (http://www.affective-sciences.org/gemep/perceived_voice). Pre-tests of the procedure showed that listeners understood the procedure and were able to use it without difficulty.

Each rater evaluated 48 recordings (2 expressions x 8 emotions x 3 speakers) on the eight voice scales described in the article and listed in Table S5 (for a total of 48 x 8 = 384 ratings). Identical computers, sound cards and headphones were used for all participants and all sessions. The scales and the speakers were presented sequentially, in a different random order for each listener. Answers were recorded by the computer on a continuous scale from 0 to 10 (no numbers were visible to the raters; labels described in the article were indicated as scale end-points).

Four groups of raters were recruited to assess the total set of vocal expressions included in this study (144 expressions produced by 9 actors). The raters in the four groups assessed the vocal expressions produced by one common speaker in order to control for group differences. No systematic differences were found across groups. In order to keep the number of ratings (and hence the reliability of the assessments) comparable across speakers we randomly removed 75% of the ratings collected for the speaker who's vocal expressions had been evaluated by all groups.

We then computed average ratings for each vocal expression (N = 144) based on either 15 or 16 ratings for each scale.

GVA corpus. A traditional rating procedure with visual analog scales was used to examine the possibility of obtaining reliable ratings at a lesser cost. The ratings were collected in eight successive blocks for separate scales (same rating scales as for the MUC corpus). Breaks were allowed in between rating blocks. The order of the blocks (i.e., scales) was randomized for each participant. The scales were shown in visual analog format (i.e., without numeric values, only labels defining the scale end points). The audio illustrations of each scale were made available on both ends of the scale. Participants were required to listen to the examples before rating the portrayals on each new scale. In each block, all samples were randomly presented by speaker. The speaker order was randomized across blocks, and the order of the blocks was randomized. A replay button was added to the bottom of the screen that allowed the vocal expressions to be replayed. Participants listened to each portrayal and then reported their answer immediately after hearing each expression on the visual-analog scale. Answers were recorded as values ranging from 0 to 100 (no numbers were shown to the raters). Identical computers, sound cards and headphones were used for all participants.

Participants provided ratings in one session of three hours. Each participant provided 160 x 8 = 1280 ratings for this part of the rating study.

Ratings of perceived emotions

MUC corpus. The ratings have not been described in earlier publications. The procedure described above to collect ratings of proximal voice cues was applied for collecting ratings of emotional intensity for four emotions (happiness, anger, fear and sadness). The vocal expressions were presented on separate screens for each speaker (with a random order for each portrayal and a random order of speakers). The task of the rater was to place the expressions on a visual analogue scale (ranging from "not emotional" to "extremely emotional"). This procedure was repeated for the four considered emotions. Each rater assessed the expression produced by three speakers. Four separate groups of raters were recruited in order to assess the expressions produced by all speakers. Average age and gender distribution of the four groups are shown in Table S5.

GVA corpus.

The description is adapted from

Bänziger T, Scherer KR. Introducing the Geneva multimodal emotion portrayal (GEMEP) corpus. In: Scherer KR, Bänziger T, Roesch EB, editors. Blueprint for affective computing: a source book. Oxford: Oxford University Press; 2010. pp. 271–294.

Ninety participants, mostly undergraduate students from different departments, including psychology, were recruited via announcements in the university buildings and outside the university. The participants were randomly assigned to rating either audio-only (31 participants, 18 female, 29 years on average), or video-only (31 participants, 25 female, 23 years on average), or audio-video portrayals (28 participants, 15 female, 29 years on average). In the current re-analysis ratings provided by the first group (rating audio-only expressions) are used. And the 160 selected portrayals are a subset of the larger corpus, which includes 1260 emotional expressions in total.

The 1,260 portrayals were rated in 10 sessions of 126 portrayals produced by separate actors. A rating session always started with a set of written instructions on the rating procedure and the definitions of the emotion categories portrayed by the actors. All sessions took place in a small laboratory equipped with six computers separated by “open space” walls. Headphones were used to display the sound. One to four raters could take part simultaneously. In each session, a computer interface displayed the portrayals produced by a selected actor in two blocks: the 96 standard sentences produced by the actor were presented first in random order (a new random order for each rater was computed at the start of each session), followed by a short break and then by the 30 portrayals produced with a sustained “aaa” by the same actor, also in random order. The intensity of the sound recordings was normalized within each block to accommodate the hearing of the raters (the actors screamed in some recordings and whispered in others; the resulting variability is so large that it would not have been possible to display all recordings at a constant sound level without normalizing the sound level beforehand). Several preset orders were defined for the successive sessions to counterbalance the sequence of actors rated. However, perfect counterbalancing was not achievable because we did not request that all raters complete the 10 rating sessions.

The ratings were collected with a computer interface, which always displayed the portrayal to be rated either in audio-only (A), in video-only (V), or in audio-video (AV) modality, depending on the randomly assigned condition for a given rater.

Instruments and procedures. First, a rating of the "believability" of each emotional portrayal was requested. Believability was rated on a continuous visual analog scale, the location of the cursor on screen being transformed to a linear scale ranging from 0 to 10. The scale was defined on screen as the "capacity of the actor to communicate a natural emotional impression" and ranged from "very low – one does not get the impression of a real emotion" to "very high – one gets the impression of a real emotion."

Upon confirmation of the rater's answer regarding believability, the computer displayed the 15 emotion categories portrayed by the actor on a circle (a variant of the Geneva Emotion Wheel; Scherer, 2005). The task of the participants was to select one or two categories on this circle and simultaneously rate the level of intensity (on a 4-point scale) for each of the selected categories. The emotional intensity was represented visually by the size of a bubble on screen. A legend specified that the smallest bubble corresponded to a "very weak emotion," a larger bubble to a "rather weak emotion," an even larger bubble to a "rather strong emotion," and the largest bubble to a "very strong emotion." The definitions of emotions were displayed on screen when the rater was moving the cursor over the respective categories (colored bubbles). The 15 categories are located on the circle according to their conceptual proximity, with positive emotions to the right side of the screen and negative emotions to the left side of the screen. Raters could select the white bubble in the center of the circle if they wished to indicate that the recording did not express an emotion. They could also click a button to type another description for the emotion portrayed in any recording (this answer was classified as "other emotion"). When a rater reported two categories, he or she had to answer a further pop-up question before proceeding to the evaluation of the next portrayal. Raters were asked to indicate if the reason for reporting two answers was either (forced choice) because those two emotions were represented in the portrayal ("mixed emotion") or because the rater was unsure and could not decide which of the two answers was "correct." The raters could replay the portrayal as often as they wished, both before rating believability and before selecting one or two categories.

For the current study the proportion of rater reporting one of the four considered categories (happiness, anger, sadness, fear) was use as assessment of perceived emotion. Those four categories comprise each two distinct categories in the rating task (elation and pleasure, hot anger and irritation, despair and sadness, panic fear and anxiety). I.e. the proportion of raters who reported either elation or pleasure was coded as the intensity of perceived happiness. The proportion of raters who reported either hot anger or irritation was coded as the intensity of perceived anger. The proportion of raters who reported either despair or sadness was coded as the intensity of perceived sadness. The proportion of raters who reported either panic fear or anxiety was coded as the intensity of perceived fear.

Table E. Raters and estimates of inter-rater agreement.

Proximal voice ratings

Corpus MUC

No. of raters (no. women)		Mean Age (*SD*)	ICC-R (averaged for the four groups)							
			rough	artic	into	insta	pitch	sharp	sp.rate	loud
Gp1	16 (14)	21.3 (4.3)	.858	.865	.907	.930	.942	.952	.967	.989
Gp2	15 (10)	21.7 (4.3)	ICC-r (averaged for the four groups)							
Gp3	15 (13)	20.2 (1.7)	rough	artic	into	insta	pitch	sharp	sp.rate	loud
Gp4	15 (11)	21.4 (6.1)	.305	.316	.395	.468	.517	.588	.661	.854

Raters in different groups assessed different subsets of portrayals from corpus M.

Corpus GVA

No. of raters (no. women)	Mean Age (*SD*)	ICC-R							
		rough	artic	into	insta	pitch	sharp	sp.rate	loud
19 (10)	22.4 (2.2)	.840	.907	.919	.934	.959	.963	.967	.988
		ICC-r							
		rough	artic	into	insta	pitch	sharp	sp.rate	loud

.216	.339	.375	.427	.554	.579	.605	.811

All raters assessed all portrayals from corpus G.

Emotion ratings

Corpus MUC

No. of raters (no. women)		Mean Age (*SD*)	ICC-R (averaged for the four groups) happiness	fear	sadness	anger
Gp1	14 (10)	21.5 (4.0)	.926	.938	.957	.966
Gp2	14 (11)	22.3 (5.7)	ICC-r (averaged for the four groups)			
Gp3	14 (12)	21.5 (3.0)	happiness	fear	sadness	anger
Gp4	14 (12)	23.3 (7.6)	.477	.529	.621	.667

Raters in different groups assessed different subsets of portrayals corpus M.

Corpus GVA

One group of 23 listeners (13 women) rated all of the portrayals, average age = 29 years (*SD* = 8.5). The inter-rater reliabilities were estimated as profile correlations, including only the categorical ratings provided for the portrayals used in this study (160 emotion portrayals and eight emotions). The average profile correlation in this sample is .763, ICC-r = .734 and ICC-R = .985.

Arousal ratings

Corpus MUC

No. of raters (no. women)	Mean Age (*SD*)		Arousal
24 (24)	21.6 (2.7)	ICC R	.979
		ICC r	.657

Corpus GVA

No. of raters (no. women)	Mean Age (*SD*)		Arousal
19 (10)	22.4 (2.2)	ICC R	.979
		ICC r	.710

Same group of raters as for proximal voice ratings. Rough = roughness, artic = articulation, into = intonation, instab = instability, sharp = sharpness, sp.rate = speech rate, loud = loudness, ICC = intra-class correlation (inter-rater consistency); ICC-r = single measure (estimate of the average correlation between all pairs of raters), ICC-R = average measure (equivalent to Cronbach's alpha).
