# Supplementary material for: Path Models of Vocal Emotion Communication
Source: PLoS One. 2015 Sep 1;10(9):e0136675. doi: 10.1371/journal.pone.0136675 (PMC4556609; doi:10.1371/journal.pone.0136675)
Supplement: S2 File — Comprehensive listing of statistical results. (PDF) [file pone.0136675.s002.pdf]

Supplemental information for

Baenziger et al. - *Path Models of Vocal Emotion Communication*

S2 – Data

(A) Detailed results and graphical illustration for the regressions included in the Lens Model Equations

Table A. Coefficients of the LME analysis.

		For the MUC data set Coefficients for expressed emotions with proximal cues			Coefficients for perceived emotions with proximal cues		
		**Beta**	***t***	**Sig.**	**Beta**	***t***	**Sig.**
anger							
	articulation	-.015	-.163	.871	.143	2.022	.045
	intonation	.144	1.170	.244	-.141	-1.463	.146
	loudness	.262	1.526	.129	.360	2.672	.008
	pitch	-.492	-3.202	.002	-.249	-2.060	.041
	roughness	.117	1.212	.227	.231	3.047	.003
	speech rate	-.091	-1.016	.311	.175	2.504	.013
	sharpness	.454	1.858	.065	.443	2.312	.022
	instability	-.486	-5.476	.000	-.148	-2.123	.036
fear							
	articulation	.398	3.562	.001	.316	3.623	.000
	intonation	-.301	-1.970	.051	-.517	-4.339	.000
	loudness	-.135	-.632	.529	-.035	-.208	.836
	pitch	.269	1.407	.162	.298	1.998	.048
	roughness	.090	.754	.452	-.052	-.552	.582
	speech rate	.823	7.428	.000	.626	7.251	.000
	sharpness	-.634	-2.090	.038	-.181	-.763	.447
	instability	.548	4.973	.000	.893	10.387	.000
happiness							
	articulation	-.217	-1.814	.072	-.032	-.317	.752
	intonation	.570	3.495	.001	.555	4.067	.000

	loudness	-.029	-.126	.900	-.094	-.493	.623
	pitch	.197	.963	.337	.457	2.672	.008
	roughness	-.172	-1.346	.181	-.217	-2.019	.045
	speech rate	-.266	-2.249	.026	-.043	-.430	.668
	sharpness	-.336	-1.035	.303	-.345	-1.270	.206
	instability	-.350	-2.968	.004	-.119	-1.203	.231
sadness							
	articulation	-.167	-1.655	.100	-.030	-.385	.701
	intonation	-.413	-3.004	.003	-.571	-5.436	.000
	loudness	-.098	-.511	.610	.029	.197	.844
	pitch	.027	.155	.877	.116	.884	.378
	roughness	-.035	-.321	.748	-.105	-1.273	.205
	speech rate	-.466	-4.666	.000	-.310	-4.064	.000
	sharpness	.516	1.887	.061	.305	1.460	.147
	instability	.287	2.890	.004	.635	8.373	.000
arousal							
	articulation	-.164	-2.047	.043	.035	.929	.355
	intonation	.339	3.093	.002	.079	1.523	.130
	loudness	.301	1.970	.051	.606	8.382	.000
	pitch	.024	.175	.861	-.037	-.567	.572
	roughness	.062	.717	.475	.110	2.709	.008
	speech rate	.086	1.085	.280	.296	7.863	.000
	sharpness	.089	.407	.685	.037	.360	.719
	instability	.226	2.856	.005	.236	6.301	.000

For the MUC data set

		Coefficients for expressed emotions with distal cues			Coefficients for perceived emotions with distal cues		
		**Beta**	***t***	**Sig.**	**Beta**	***t***	**Sig.**
anger							
	F0 floor (either min or 5th percentile)	-.521	-6.943	.000	-.136	-1.994	.048
	F0 range	-.122	-1.411	.160	-.082	-1.046	.298
	intensity mean	.643	4.497	.000	.656	5.064	.000

	intensity range	-.058	-.800	.425	.060	.901	.369
	acoustic duration	.079	1.068	.287	.010	.152	.880
	relative voiced duration	-.030	-.379	.705	-.038	-.528	.598
	relative energy 600-800	-.119	-1.761	.080	-.145	-2.372	.019
	relative energy < 1000	-.349	-3.791	.000	-.289	-3.453	.001
fear							
	F0 floor (either min or 5th percentile)	.316	3.613	.000	.550	6.519	.000
	F0 range	-.120	-1.196	.234	-.043	-.442	.659
	intensity mean	.064	.383	.702	.008	.052	.959
	intensity range	.226	2.662	.009	.207	2.518	.013
	acoustic duration	-.305	-3.526	.001	-.112	-1.345	.181
	relative voiced duration	-.266	-2.856	.005	-.151	-1.686	.094
	relative energy 600-800	-.199	-2.537	.012	-.077	-1.022	.309
	relative energy < 1000	.337	3.138	.002	.304	2.929	.004
happiness							
	F0 floor (either min or 5th percentile)	-.041	-.399	.691	.111	1.134	.259
	F0 range	.173	1.476	.142	.227	2.019	.045
	intensity mean	-.309	-1.584	.115	-.035	-.190	.850
	intensity range	.026	.265	.792	.182	1.911	.058
	acoustic duration	-.155	-1.530	.128	-.197	-2.031	.044
	relative voiced duration	.074	.678	.499	-.042	-.407	.685
	relative energy 600-800	.156	1.702	.091	.117	1.332	.185
	relative energy < 1000	.003	.021	.983	.139	1.157	.249
sadness							
	F0 floor (either min or 5th percentile)	.246	2.796	.006	.439	5.614	.000
	F0 range	.068	.675	.501	.100	1.117	.266
	intensity mean	-.398	-2.373	.019	-.415	-2.786	.006
	intensity range	-.194	-2.269	.025	-.031	-.414	.679
	acoustic duration	.380	4.368	.000	.322	4.160	.000
	relative voiced duration	.222	2.371	.019	.163	1.962	.052
	relative energy 600-800	.161	2.042	.043	.119	1.701	.091
	relative energy < 1000	.010	.092	.927	.282	2.935	.004
arousal							

F0 floor (either min or 5th percentile)	.365	5.489	.000	.167	3.488	.001
F0 range	.241	3.164	.002	.137	2.497	.014
intensity mean	.230	1.817	.071	.590	6.476	.000
intensity range	.216	3.340	.001	.194	4.169	.000
acoustic duration	-.053	-.813	.418	-.073	-1.534	.127
relative voiced duration	-.055	-.784	.434	-.150	-2.943	.004
relative energy 600-800	-.005	-.090	.929	-.081	-1.895	.060
relative energy < 1000	-.036	-.443	.658	-.116	-1.974	.050

		**For the GVA data set**					
		**Coefficients for expressed emotions with proximal cues**			**Coefficients for perceived emotions with proximal cues**		
		Beta	*t*	Sig.	Beta	*t*	Sig.
anger							
	articulation	.074	.845	.400	.133	1.847	.067
	intonation	.302	3.236	.001	.077	1.009	.315
	loudness	.229	1.093	.276	.258	1.503	.135
	pitch	-.090	-.625	.533	-.171	-1.442	.151
	roughness	.198	2.362	.019	.266	3.871	.000
	speech rate	-.055	-.760	.449	.004	.064	.949
	sharpness	-.020	-.090	.928	.345	1.898	.060
	instability	-.684	-8.509	.000	-.520	-7.886	.000
fear							
	articulation	.174	1.510	.133	.319	3.306	.001
	intonation	-.242	-1.981	.049	-.367	-3.583	.000
	loudness	.152	.556	.579	.097	.421	.674
	pitch	-.166	-.881	.380	-.034	-.214	.831
	roughness	-.110	-1.005	.317	-.053	-.573	.568
	speech rate	.654	6.863	.000	.704	8.802	.000
	sharpness	-.194	-.669	.504	-.167	-.687	.493
	instability	.486	4.621	.000	.806	9.136	.000
happiness							
	articulation	-.182	-1.478	.141	-.126	-1.077	.283

	intonation	.326	2.500	.014	.436	3.506	.001
	loudness	-.546	-1.866	.064	-.736	-2.638	.009
	pitch	.087	.430	.668	.141	.733	.465
	roughness	-.104	-.888	.376	-.118	-1.052	.294
	speech rate	-.495	-4.855	.000	-.423	-4.353	.000
	sharpness	.402	1.297	.197	.448	1.515	.132
	instability	-.148	-1.317	.190	-.117	-1.092	.276
sadness							
	articulation	-.066	-.506	.614	-.236	-2.079	.039
	intonation	-.386	-2.784	.006	-.591	-4.910	.000
	loudness	.165	.532	.596	.700	2.594	.010
	pitch	.170	.791	.430	.119	.639	.524
	roughness	.016	.131	.896	-.133	-1.226	.222
	speech rate	-.104	-.961	.338	-.247	-2.628	.009
	sharpness	-.188	-.570	.569	-.433	-1.515	.132
	instability	.346	2.894	.004	.518	5.000	.000
arousal							
	articulation	.106	1.634	.104	-.010	-.269	.788
	intonation	-.044	-.640	.523	.133	3.286	.001
	loudness	.305	1.985	.049	.462	5.074	.000
	pitch	-.023	-.220	.826	-.060	-.949	.344
	roughness	.013	.208	.836	.071	1.958	.052
	speech rate	.026	.490	.625	.107	3.377	.001
	sharpness	.551	3.381	.001	.413	4.282	.000
	instability	.321	5.436	.000	.091	2.611	.010

For the GVA data set

		**Coefficients for expressed emotions with distal cues**			**Coefficients for perceived emotions with distal cues**		
		Beta	*t*	Sig.	Beta	*t*	Sig.
anger							
	F0 floor (either min or 5th percentile)	-.660	-5.532	.000	-.699	-6.687	.000
	F0 range	-.080	-.939	.349	-.152	-2.022	.045

	intensity mean	.730	4.201	.000	.833	5.469	.000
	intensity range	.055	.554	.581	.034	.395	.693
	acoustic duration	.095	1.164	.246	.079	1.112	.268
	relative voiced duration	-.002	-.020	.984	.034	.464	.644
	relative energy 600-800	-.109	-1.149	.253	-.095	-1.143	.255
	relative energy < 1000	-.162	-1.339	.183	-.351	-3.321	.001
fear							
	F0 floor (either min or 5th percentile)	.287	2.340	.021	.461	3.990	.000
	F0 range	-.120	-1.368	.173	-.006	-.070	.945
	intensity mean	-.229	-1.281	.202	-.263	-1.560	.121
	intensity range	.347	3.388	.001	.301	3.115	.002
	acoustic duration	-.383	-4.569	.000	-.416	-5.264	.000
	relative voiced duration	-.133	-1.560	.121	-.224	-2.800	.006
	relative energy 600-800	-.058	-.592	.555	.065	.709	.479
	relative energy < 1000	-.024	-.190	.850	.105	.895	.372
happiness							
	F0 floor (either min or 5th percentile)	.171	1.433	.154	.382	3.227	.002
	F0 range	.073	.850	.397	.223	2.630	.009
	intensity mean	.033	.190	.849	-.138	-.799	.426
	intensity range	-.373	-3.738	.000	-.318	-3.220	.002
	acoustic duration	.486	5.950	.000	.409	5.060	.000
	relative voiced duration	.113	1.366	.174	.136	1.654	.100
	relative energy 600-800	-.097	-1.024	.307	-.091	-.964	.337
	relative energy < 1000	.099	.822	.412	.210	1.756	.081
sadness							
	F0 floor (either min or 5th percentile)	.201	1.579	.116	.294	2.312	.022
	F0 range	.128	1.399	.164	.036	.395	.694
	intensity mean	-.534	-2.876	.005	-.515	-2.784	.006
	intensity range	-.029	-.275	.784	-.017	-.162	.871
	acoustic duration	-.198	-2.270	.025	-.122	-1.410	.161
	relative voiced duration	.021	.240	.810	.005	.055	.956
	relative energy 600-800	.264	2.605	.010	.316	3.132	.002
	relative energy < 1000	.086	.664	.507	.147	1.144	.255

arousal							
	F0 floor (either min or 5th percentile)	.257	4.112	.000	.126	2.994	.003
	F0 range	.084	1.878	.062	.123	4.071	.000
	intensity mean	.617	6.783	.000	.613	10.000	.000
	intensity range	-.053	-1.023	.308	.055	1.571	.118
	acoustic duration	.049	1.158	.249	-.012	-.426	.671
	relative voiced duration	.016	.376	.707	-.006	-.194	.847
	relative energy 600-800	.056	1.124	.263	.038	1.150	.252
	relative energy < 1000	-.003	-.042	.966	-.140	-3.294	.001

Fig. A. Application of the LME approach to the vocal communication of anger in the MUC corpus.

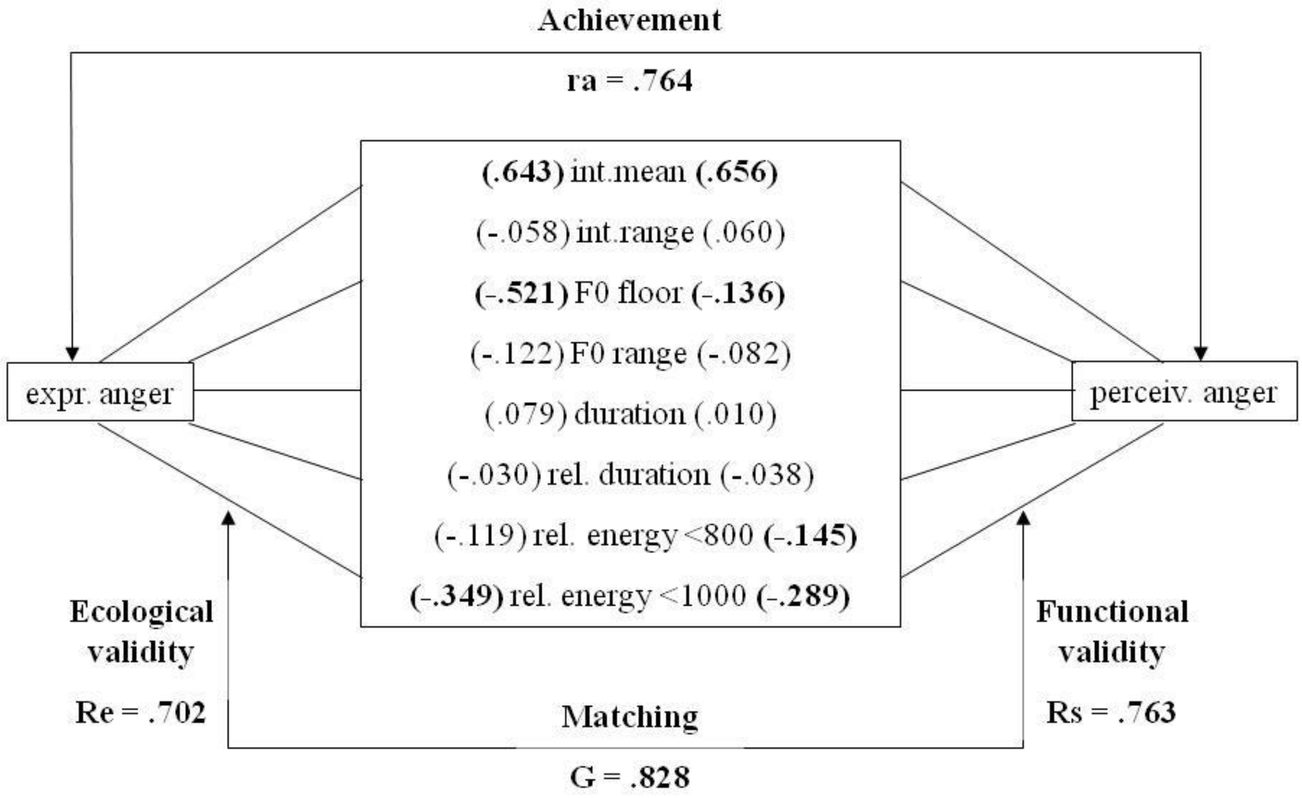

(a) Model based on eight distal cues (acoustic measures). Beta coefficients in parentheses and significant coefficients ($p < .05$) in bold. int. = intensity; rel. = relative; expr. = expressed; perceiv. = perceived.

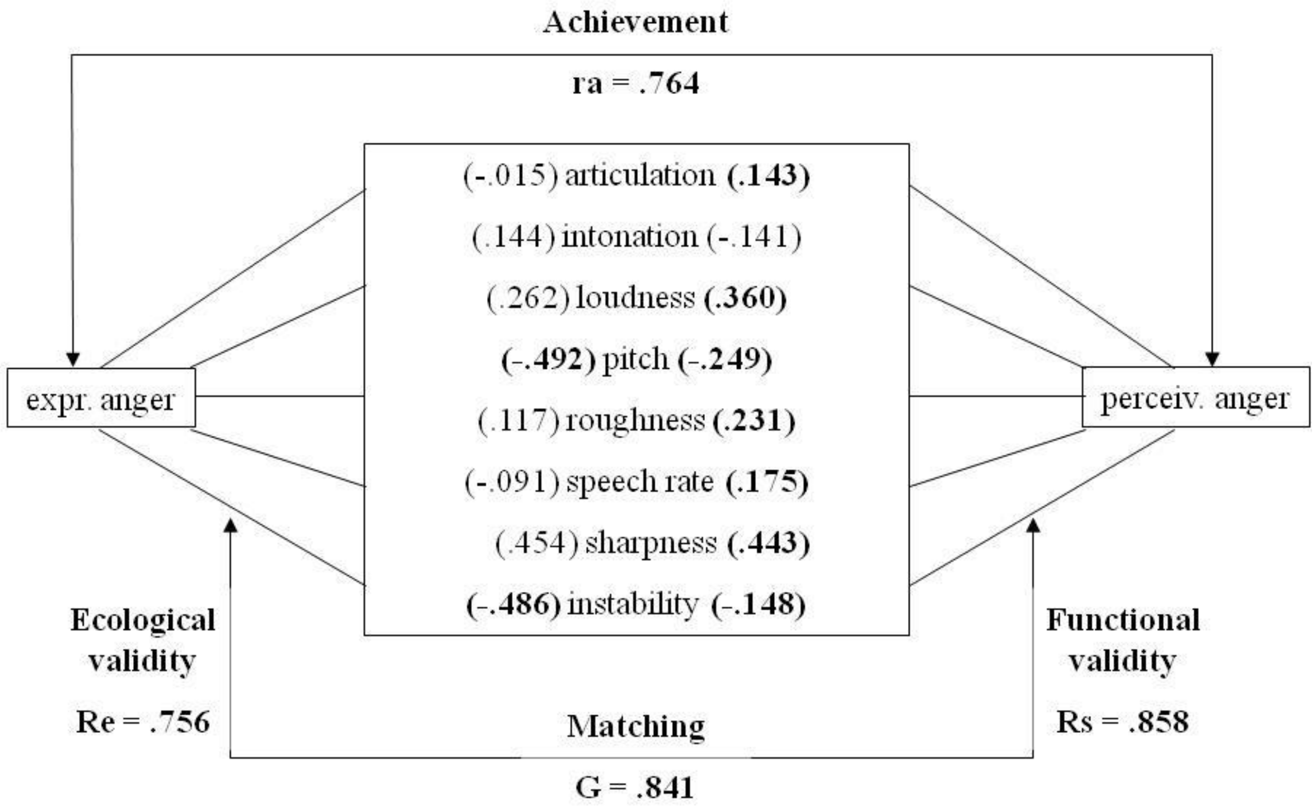

(b) Model based on eight proximal cues (perceived voice features). Beta coefficients in parentheses and significant coefficients ($p < .05$) in bold. expr. = expressed; perceiv. = perceived.

(B) Complete design for path analysis

Table B. Mplus instruction statement for the path analysis.

This syntax was used for computing the results in Tables 4, 5 and 6.

```
Mplus VERSION 6.1

MUTHEN & MUTHEN

12/15/2014   6:53 PM


INPUT INSTRUCTIONS


  Data:

      File is data_mplus_2710.txt;

  Variable:

      Names are

      anger_t happy_t fear_t sad_t arou_t

      anger_p happy_p fear_p sad_p arou_p

      anger_as happy_as fear_as sad_as arou_as

      zint_m zint_rg zf0_05 zf0_rg zdur zrelen

      intona loud pitch rough sprate instab;


      usevariables are

      anger_t fear_t sad_t arou_t

      anger_as happy_as fear_as sad_as arou_as

      intona loud pitch rough sprate instab

      zint_m zint_rg zf0_05 zf0_rg zdur zrelen;


      missing is all (-9);
```

```
Analysis:   estimator=MLR;
           !bootstrap=1000;

Model:
anger_as on intona-instab zint_m-zrelen anger_t sad_t fear_t arou_t;
happy_as on intona-instab zint_m-zrelen anger_t sad_t fear_t arou_t;
fear_as on intona-instab zint_m-zrelen fear_t anger_t sad_t fear_t arou_t;
sad_as on intona-instab zint_m-zrelen sad_t anger_t sad_t fear_t arou_t;
arou_as on intona-instab zint_m-zrelen sad_t anger_t sad_t fear_t arou_t;

intona on zint_m-zrelen anger_t sad_t fear_t arou_t;
loud on zint_m-zrelen anger_t sad_t fear_t arou_t;
pitch on zint_m-zrelen anger_t sad_t fear_t arou_t;
rough on zint_m-zrelen anger_t sad_t fear_t arou_t;
sprate on zint_m-zrelen anger_t sad_t fear_t arou_t;
instab on zint_m-zrelen anger_t sad_t fear_t arou_t;

zint_m on anger_t sad_t fear_t arou_t;
zint_rg on anger_t sad_t fear_t arou_t;
zf0_05 on anger_t sad_t fear_t arou_t;
zf0_rg on anger_t sad_t fear_t arou_t;
zdur on anger_t sad_t fear_t arou_t;
zrelen on anger_t sad_t fear_t arou_t;

intona with loud-instab;
loud with pitch-instab;
pitch with rough-instab;
rough with sprate-instab;
sprate with instab;
```

```
zint_m with zint_rg-zrelen;
zint_rg with zf0_05-zrelen;
zf0_05 with zf0_rg-zrelen;
zf0_rg with zdur-zrelen;
zdur with zrelen;

Output: stdyx modindices;
!cinterval(bcbootstrap);

Model indirect:
anger_as ind anger_t;
fear_as ind fear_t;
sad_as ind sad_t;
arou_as ind arou_t;
```

Note. Anger_t = expressed anger

Fear_t = expressed fear

Sad_t = expressed sadness

Arou_t = expressed arousal

anger_as = perceived anger (square-root-arcussinus transformed)

happy_as = perceived happiness (square-root-arcussinus transformed)

fear_as = perceived fear (square-root-arcussinus transformed)

sad_as = perceived sadness (square-root-arcussinus transformed)

intona = intonation

loud = loudness

pitch = pitch

rough = roughness

sprate = speech rate

instab = instability

zint_mean = intensity mean

zint_rg = intensity range

zf0_05 = F0 floor

zf0_rg = F0 range

zdur = duration

zrelen = relative energy

(C) Complete results for path analysis

Table C. Mplus output (standardized results only).

This is the Mplus output that was used for creating Tables 4, 5 and 6.

STANDARDIZED MODEL RESULTS

STDYX Standardization

	Estimate	S.E.	Est./S.E.	Two-tailed *P*-Value
ANGER_AS ON				
INTONA	-0.043	0.058	-0.746	0.456
LOUD	0.296	0.093	3.190	0.001
PITCH	-0.039	0.069	-0.569	0.570
ROUGH	0.177	0.039	4.547	0.000
SPRATE	-0.037	0.065	-0.563	0.573
INSTAB	-0.257	0.049	-5.273	0.000
ZINT_M	-0.033	0.086	-0.381	0.703
ZINT_RG	0.039	0.038	1.013	0.311
ZF0_05	0.107	0.047	2.264	0.024
ZF0_RG	0.025	0.042	0.603	0.546
ZDUR	0.032	0.048	0.663	0.507
ZRELEN	0.000	0.044	-0.005	0.996
ANGER_T	0.566	0.050	11.389	0.000
SAD_T	0.082	0.043	1.881	0.060
FEAR_T	0.145	0.045	3.227	0.001
AROU_T	0.103	0.052	1.988	0.047
HAPPY_AS ON				
INTONA	0.205	0.080	2.578	0.010

LOUD	-0.242	0.132	-1.840	0.066
PITCH	0.201	0.090	2.241	0.025
ROUGH	0.056	0.046	1.216	0.224
SPRATE	0.009	0.072	0.124	0.902
INSTAB	-0.073	0.063	-1.157	0.247
ZINT_M	0.064	0.116	0.547	0.584
ZINT_RG	0.026	0.053	0.493	0.622
ZF0_05	0.025	0.061	0.409	0.682
ZF0_RG	0.015	0.051	0.297	0.767
ZDUR	0.030	0.059	0.504	0.614
ZRELEN	0.052	0.067	0.775	0.438
ANGER_T	-0.717	0.062	-11.555	0.000
SAD_T	-0.654	0.051	-12.756	0.000
FEAR_T	-0.652	0.059	-11.003	0.000
AROU_T	-0.004	0.080	-0.050	0.960
FEAR_AS ON				
INTONA	-0.124	0.067	-1.848	0.065
LOUD	-0.051	0.104	-0.493	0.622
PITCH	0.070	0.078	0.897	0.370
ROUGH	-0.054	0.044	-1.229	0.219
SPRATE	0.168	0.063	2.659	0.008
INSTAB	0.356	0.058	6.152	0.000
ZINT_M	0.099	0.104	0.957	0.339
ZINT_RG	0.055	0.046	1.207	0.228
ZF0_05	0.014	0.052	0.272	0.785
ZF0_RG	-0.039	0.047	-0.824	0.410
ZDUR	-0.039	0.054	-0.726	0.468
ZRELEN	0.000	0.050	-0.004	0.997
FEAR_T	0.668	0.046	14.596	0.000

ANGER_T	0.130	0.052	2.509	0.012
SAD_T	0.257	0.049	5.203	0.000
AROU_T	-0.004	0.059	-0.060	0.953
SAD_AS ON				
INTONA	-0.208	0.073	-2.841	0.004
LOUD	0.284	0.112	2.529	0.011
PITCH	0.110	0.074	1.471	0.141
ROUGH	-0.039	0.048	-0.801	0.423
SPRATE	-0.211	0.066	-3.172	0.002
INSTAB	0.279	0.060	4.612	0.000
ZINT_M	-0.063	0.108	-0.590	0.555
ZINT_RG	0.003	0.043	0.071	0.943
ZF0_05	-0.022	0.054	-0.416	0.678
ZF0_RG	-0.023	0.052	-0.431	0.666
ZDUR	-0.122	0.056	-2.187	0.029
ZRELEN	0.077	0.056	1.373	0.170
SAD_T	0.630	0.049	12.780	0.000
ANGER_T	-0.006	0.053	-0.113	0.910
FEAR_T	0.164	0.053	3.074	0.002
AROU_T	-0.023	0.067	-0.337	0.736
AROU_AS ON				
INTONA	0.070	0.041	1.716	0.086
LOUD	0.681	0.053	12.956	0.000
PITCH	-0.041	0.034	-1.200	0.230
ROUGH	0.042	0.019	2.150	0.032
SPRATE	0.119	0.033	3.645	0.000
INSTAB	0.181	0.034	5.339	0.000
ZINT_M	-0.124	0.054	-2.305	0.021

ZINT_RG	-0.016	0.021	-0.780	0.435
ZF0_05	0.069	0.026	2.628	0.009
ZF0_RG	0.063	0.025	2.556	0.011
ZDUR	0.012	0.028	0.442	0.659
ZRELEN	-0.078	0.032	-2.456	0.014
SAD_T	-0.060	0.019	-3.123	0.002
ANGER_T	0.105	0.030	3.555	0.000
FEAR_T	0.074	0.024	3.082	0.002
AROU_T	0.125	0.028	4.379	0.000
INTONA ON				
ZINT_M	0.305	0.082	3.703	0.000
ZINT_RG	0.127	0.048	2.650	0.008
ZF0_05	0.176	0.049	3.586	0.000
ZF0_RG	0.288	0.045	6.451	0.000
ZDUR	-0.033	0.047	-0.699	0.484
ZRELEN	0.071	0.048	1.492	0.136
ANGER_T	-0.180	0.050	-3.594	0.000
SAD_T	-0.252	0.040	-6.237	0.000
FEAR_T	-0.187	0.042	-4.420	0.000
AROU_T	0.172	0.060	2.853	0.004
LOUD ON				
ZINT_M	0.752	0.058	13.055	0.000
ZINT_RG	0.095	0.024	3.920	0.000
ZF0_05	0.007	0.029	0.241	0.810
ZF0_RG	0.010	0.026	0.392	0.695
ZDUR	0.025	0.025	0.987	0.324
ZRELEN	-0.082	0.031	-2.597	0.009
ANGER_T	0.077	0.024	3.149	0.002

SAD_T	-0.001	0.023	-0.023	0.981
FEAR_T	0.029	0.022	1.286	0.199
AROU_T	0.068	0.038	1.781	0.075
PITCH ON				
ZINT_M	0.388	0.093	4.189	0.000
ZINT_RG	0.059	0.048	1.212	0.225
ZF0_05	0.374	0.060	6.203	0.000
ZF0_RG	0.322	0.052	6.179	0.000
ZDUR	-0.063	0.047	-1.353	0.176
ZRELEN	0.077	0.066	1.168	0.243
ANGER_T	-0.325	0.051	-6.349	0.000
SAD_T	-0.085	0.038	-2.249	0.025
FEAR_T	-0.096	0.040	-2.425	0.015
AROU_T	-0.013	0.065	-0.201	0.841
ROUGH ON				
ZINT_M	-0.222	0.126	-1.764	0.078
ZINT_RG	-0.167	0.067	-2.492	0.013
ZF0_05	-0.189	0.086	-2.200	0.028
ZF0_RG	0.024	0.072	0.329	0.742
ZDUR	0.167	0.065	2.556	0.011
ZRELEN	-0.243	0.088	-2.758	0.006
ANGER_T	0.346	0.078	4.452	0.000
SAD_T	0.224	0.068	3.296	0.001
FEAR_T	0.171	0.064	2.676	0.007
AROU_T	0.295	0.096	3.080	0.002
SPRATE ON				
ZINT_M	0.190	0.086	2.199	0.028

ZINT_RG	0.020	0.043	0.474	0.636
ZF0_05	0.034	0.052	0.647	0.518
ZF0_RG	0.104	0.042	2.465	0.014
ZDUR	-0.542	0.038	-14.371	0.000
ZRELEN	-0.114	0.054	-2.092	0.036
ANGER_T	0.076	0.051	1.488	0.137
SAD_T	-0.029	0.035	-0.844	0.398
FEAR_T	0.289	0.044	6.559	0.000
AROU_T	0.089	0.056	1.595	0.111
INSTAB ON				
ZINT_M	-0.071	0.091	-0.780	0.435
ZINT_RG	0.014	0.049	0.279	0.780
ZF0_05	0.174	0.054	3.209	0.001
ZF0_RG	0.181	0.048	3.737	0.000
ZDUR	0.223	0.044	5.046	0.000
ZRELEN	0.063	0.054	1.155	0.248
ANGER_T	-0.421	0.054	-7.834	0.000
SAD_T	0.274	0.044	6.220	0.000
FEAR_T	0.184	0.049	3.756	0.000
AROU_T	0.214	0.069	3.094	0.002
ZINT_M ON				
ANGER_T	0.340	0.037	9.287	0.000
SAD_T	-0.135	0.032	-4.172	0.000
FEAR_T	0.046	0.031	1.462	0.144
AROU_T	0.766	0.027	28.725	0.000
ZINT_RG ON				
ANGER_T	0.160	0.056	2.839	0.005

SAD_T	-0.089	0.055	-1.626	0.104
FEAR_T	0.123	0.059	2.096	0.036
AROU_T	0.519	0.040	13.032	0.000
ZF0_05 ON				
ANGER_T	-0.020	0.048	-0.408	0.684
SAD_T	0.016	0.048	0.322	0.748
FEAR_T	0.195	0.051	3.853	0.000
AROU_T	0.684	0.030	22.524	0.000
ZF0_RG ON				
ANGER_T	0.148	0.060	2.449	0.014
SAD_T	-0.110	0.057	-1.928	0.054
FEAR_T	-0.148	0.060	-2.460	0.014
AROU_T	0.477	0.044	10.949	0.000
ZDUR ON				
ANGER_T	-0.176	0.066	-2.670	0.008
SAD_T	-0.041	0.076	-0.539	0.590
FEAR_T	-0.283	0.063	-4.465	0.000
AROU_T	-0.032	0.055	-0.575	0.565
ZRELEN ON				
ANGER_T	-0.317	0.046	-6.910	0.000
SAD_T	0.108	0.040	2.712	0.007
FEAR_T	-0.007	0.048	-0.154	0.878
AROU_T	-0.619	0.038	-16.424	0.000
INTONA WITH				
LOUD	0.097	0.066	1.480	0.139

PITCH	0.524	0.048	10.856	0.000
ROUGH	-0.258	0.055	-4.673	0.000
SPRATE	0.083	0.059	1.397	0.162
INSTAB	0.256	0.058	4.379	0.000
LOUD WITH				
PITCH	0.132	0.069	1.923	0.054
ROUGH	0.068	0.074	0.922	0.357
SPRATE	0.149	0.048	3.123	0.002
INSTAB	-0.094	0.066	-1.419	0.156
PITCH WITH				
ROUGH	-0.478	0.054	-8.823	0.000
SPRATE	0.234	0.056	4.145	0.000
INSTAB	0.174	0.061	2.859	0.004
ROUGH WITH				
SPRATE	-0.163	0.053	-3.095	0.002
INSTAB	0.243	0.058	4.195	0.000
SPRATE WITH				
INSTAB	-0.044	0.056	-0.799	0.424
ZINT_M WITH				
ZINT_RG	0.117	0.065	1.802	0.072
ZF0_05	0.319	0.057	5.593	0.000
ZF0_RG	0.205	0.066	3.120	0.002
ZDUR	-0.215	0.053	-4.094	0.000
ZRELEN	-0.403	0.065	-6.247	0.000

```
 ZINT_RG  WITH
    ZF0_05             0.087      0.065      1.345      0.179
    ZF0_RG             0.124      0.059      2.115      0.034
    ZDUR               0.351      0.057      6.128      0.000
    ZRELEN            -0.004      0.058     -0.066      0.948

 ZF0_05   WITH
    ZF0_RG            -0.129      0.066     -1.973      0.048
    ZDUR               0.115      0.054      2.148      0.032
    ZRELEN            -0.309      0.053     -5.882      0.000

 ZF0_RG   WITH
    ZDUR               0.097      0.061      1.595      0.111
    ZRELEN            -0.111      0.065     -1.725      0.084

 ZDUR     WITH
    ZRELEN             0.052      0.059      0.894      0.371

 HAPPY_AS WITH
    ANGER_AS           0.231      0.054      4.242      0.000

 FEAR_AS  WITH
    ANGER_AS           0.147      0.066      2.236      0.025
    HAPPY_AS           0.117      0.053      2.221      0.026

 SAD_AS   WITH
    ANGER_AS           0.106      0.057      1.849      0.064
    HAPPY_AS           0.270      0.051      5.269      0.000
    FEAR_AS            0.141      0.062      2.275      0.023
```

AROU_AS WITH

ANGER_AS	0.154	0.055	2.809	0.005
HAPPY_AS	-0.050	0.054	-0.931	0.352
FEAR_AS	-0.123	0.051	-2.416	0.016
SAD_AS	-0.181	0.052	-3.513	0.000

Intercepts

ANGER_AS	0.650	0.091	7.131	0.000
HAPPY_AS	2.330	0.118	19.793	0.000
FEAR_AS	0.811	0.106	7.623	0.000
SAD_AS	0.911	0.113	8.036	0.000
AROU_AS	2.890	0.094	30.696	0.000
INTONA	0.185	0.094	1.959	0.050
LOUD	-0.129	0.049	-2.616	0.009
PITCH	0.305	0.090	3.402	0.001
ROUGH	-0.723	0.149	-4.847	0.000
SPRATE	-0.283	0.086	-3.287	0.001
INSTAB	-0.235	0.110	-2.140	0.032
ZINT_M	-0.911	0.054	-16.833	0.000
ZINT_RG	-0.630	0.092	-6.847	0.000
ZF0_05	-0.795	0.080	-9.935	0.000
ZF0_RG	-0.414	0.104	-3.993	0.000
ZDUR	0.320	0.129	2.487	0.013
ZRELEN	0.744	0.067	11.051	0.000

Residual Variances

ANGER_AS	0.238	0.022	10.640	0.000
HAPPY_AS	0.380	0.031	12.397	0.000
FEAR_AS	0.287	0.025	11.583	0.000
SAD_AS	0.340	0.032	10.710	0.000

AROU_AS	0.075	0.010	7.839	0.000
INTONA	0.315	0.031	10.027	0.000
LOUD	0.081	0.009	8.802	0.000
PITCH	0.303	0.030	10.004	0.000
ROUGH	0.840	0.038	21.896	0.000
SPRATE	0.289	0.030	9.581	0.000
INSTAB	0.384	0.031	12.540	0.000
ZINT_M	0.252	0.025	10.106	0.000
ZINT_RG	0.679	0.042	16.093	0.000
ZF0_05	0.492	0.041	11.990	0.000
ZF0_RG	0.702	0.048	14.532	0.000
ZDUR	0.932	0.026	35.291	0.000
ZRELEN	0.484	0.044	10.938	0.000

R-SQUARE

Observed Variable	Estimate	S.E.	Est./S.E.	Two-Tailed P-Value
ANGER_AS	0.762	0.022	34.020	0.000
HAPPY_AS	0.620	0.031	20.206	0.000
FEAR_AS	0.713	0.025	28.823	0.000
SAD_AS	0.660	0.032	20.787	0.000
AROU_AS	0.925	0.010	96.849	0.000
INTONA	0.685	0.031	21.787	0.000
LOUD	0.919	0.009	100.110	0.000
PITCH	0.697	0.030	22.963	0.000
ROUGH	0.160	0.038	4.166	0.000
SPRATE	0.711	0.030	23.572	0.000
INSTAB	0.616	0.031	20.121	0.000
ZINT_M	0.748	0.025	29.947	0.000

```
    ZINT_RG            0.321      0.042      7.616      0.000

    ZF0_05             0.508      0.041     12.371      0.000

    ZF0_RG             0.298      0.048      6.173      0.000

    ZDUR               0.068      0.026      2.574      0.010

    ZRELEN             0.516      0.044     11.676      0.000


QUALITY OF NUMERICAL RESULTS

Condition Number for the Information Matrix: 0.151E-05 (ratio of smallest to largest
eigenvalue).
```

Note. Anger_t = expressed anger

Fear_t = expressed fear

Sad_t = expressed sadness

Arou_t = expressed arousal

anger_as = perceived anger (square-root-arcussinus transformed)

happy_as = perceived happiness (square-root-arcussinus transformed)

fear_as = perceived fear (square-root-arcussinus transformed)

sad_as = perceived sadness (square-root-arcussinus transformed)

intona = intonation

loud = loudness

pitch = pitch

rough = roughness

sprate = speech rate

instab = instability

zint_mean = intensity mean

zint_rg = intensity range

zf0_05 = F0 floor

zf0_rg = F0 range

zdur = duration

zrelen = relative energy

(D) Complete design for path analysis (happiness)

The results of this analysis are not described in the article. The model described here contrasts happiness (with low and high arousal) to all other emotions.

Table D. Mplus instruction statement for a model with expressed happiness at input.

```
Data:

    File is data_mplus_2710.txt;

Variable:

    Names are

    anger_t happy_t fear_t sad_t arou_t

    anger_p happy_p fear_p sad_p arou_p

    anger_as happy_as fear_as sad_as arou_as

    zint_m zint_rg zf0_05 zf0_rg zdur zrelen

    intona loud pitch rough sprate instab;



   usevariables are

   happy_t arou_t

   happy_as

   intona loud pitch rough sprate instab

   zint_m zint_rg zf0_05 zf0_rg zdur zrelen;


    missing is all (-9);


Analysis: estimator=MLR;


Model:

    happy_as on intona-instab zint_m-zrelen happy_t arou_t;
```

```
intona on zint_m-zrelen happy_t arou_t;
loud on zint_m-zrelen happy_t arou_t;
pitch on zint_m-zrelen happy_t arou_t;
rough on zint_m-zrelen happy_t arou_t;
sprate on zint_m-zrelen happy_t arou_t;
instab on zint_m-zrelen happy_t arou_t;

zint_m on happy_t arou_t;
zint_rg on happy_t arou_t;
zf0_05 on happy_t arou_t;
zf0_rg on happy_t arou_t;
zdur on happy_t arou_t;
zrelen on happy_t arou_t;

intona with loud-instab;
loud with pitch-instab;
pitch with rough-instab;
rough with sprate-instab;
sprate with instab;

zint_m with zint_rg-zrelen;
zint_rg with zf0_05-zrelen;
zf0_05 with zf0_rg-zrelen;
zf0_rg with zdur-zrelen;
zdur with zrelen;
```

```
Output: stdyx modindices;

  Model indirect:
  happy_as ind happy_t;
```

Note. Anger_t = expressed anger

Fear_t = expressed fear

Sad_t = expressed sadness

Arou_t = expressed arousal

anger_as = perceived anger (square-root-arcussinus transformed)

happy_as = perceived happiness (square-root-arcussinus transformed)

fear_as = perceived fear (square-root-arcussinus transformed)

sad_as = perceived sadness (square-root-arcussinus transformed)

intona = intonation

loud = loudness

pitch = pitch

rough = roughness

sprate = speech rate

instab = instability

zint_mean = intensity mean

zint_rg = intensity range

zf0_05 = F0 floor

zf0_rg = F0 range

zdur = duration

zrelen = relative energy

(E) Complete results for path analysis (happiness)

Table E. Mplus output for the model with expressed happiness at input (standardized results only).

STDYX Standardization

	Estimate	S.E.	Est./S.E.	Two-Tailed P-Value
HAPPY_AS ON				
**INTONA**	**0.185**	**0.078**	**2.380**	**0.017**
LOUD	-0.245	0.131	-1.868	0.062
**PITCH**	**0.210**	**0.087**	**2.411**	**0.016**
ROUGH	0.047	0.046	1.029	0.303
SPRATE	0.015	0.068	0.223	0.824
INSTAB	-0.038	0.055	-0.690	0.490
ZINT_M	0.040	0.114	0.350	0.727
ZINT_RG	0.027	0.052	0.523	0.601
ZF0_05	0.033	0.058	0.566	0.571
ZF0_RG	0.011	0.051	0.211	0.833
ZDUR	0.021	0.059	0.355	0.723
ZRELEN	0.058	0.066	0.881	0.379
HAPPY_T	0.675	0.032	21.103	0.000
AROU_T	0.011	0.076	0.143	0.886
INTONA ON				
ZINT_M	0.346	0.072	4.828	0.000

ZINT_RG	0.142	0.046	3.055	0.002
ZF0_05	0.171	0.043	3.972	0.000
ZF0_RG	0.285	0.044	6.432	0.000
ZDUR	-0.041	0.046	-0.898	0.369
ZRELEN	0.062	0.046	1.341	0.180
HAPPY_T	0.212	0.034	6.286	0.000
AROU_T	0.131	0.057	2.306	0.021
LOUD ON				
ZINT_M	0.806	0.044	18.354	0.000
ZINT_RG	0.102	0.025	4.148	0.000
ZF0_05	-0.014	0.026	-0.537	0.591
ZF0_RG	0.009	0.026	0.366	0.714
ZDUR	0.027	0.024	1.167	0.243
ZRELEN	-0.098	0.034	-2.891	0.004
HAPPY_T	-0.031	0.017	-1.823	0.068
AROU_T	0.027	0.032	0.856	0.392
PITCH ON				
ZINT_M	0.199	0.086	2.299	0.021
ZINT_RG	0.055	0.049	1.133	0.257
ZF0_05	0.471	0.055	8.534	0.000
ZF0_RG	0.321	0.057	5.626	0.000
ZDUR	-0.093	0.044	-2.107	0.035
ZRELEN	0.139	0.067	2.060	0.039
HAPPY_T	0.159	0.031	5.111	0.000
AROU_T	0.105	0.058	1.808	0.071

ROUGH ON				
ZINT_M	-0.111	0.109	-1.021	0.307
ZINT_RG	-0.179	0.066	-2.688	0.007
ZF0_05	-0.262	0.074	-3.536	0.000
ZF0_RG	0.028	0.075	0.369	0.712
ZDUR	0.198	0.062	3.216	0.001
ZRELEN	-0.282	0.087	-3.252	0.001
HAPPY_T	-0.246	0.053	-4.623	0.000
AROU_T	0.241	0.089	2.712	0.007
SPRATE ON				
ZINT_M	0.191	0.075	2.545	0.011
ZINT_RG	0.095	0.046	2.073	0.038
ZF0_05	0.117	0.050	2.322	0.020
ZF0_RG	0.088	0.043	2.055	0.040
ZDUR	-0.616	0.036	-17.132	0.000
ZRELEN	-0.096	0.053	-1.806	0.071
HAPPY_T	-0.090	0.031	-2.887	0.004
AROU_T	0.009	0.061	0.147	0.883
INSTAB ON				
ZINT_M	-0.603	0.087	-6.954	0.000
ZINT_RG	-0.010	0.063	-0.159	0.874
ZF0_05	0.431	0.065	6.687	0.000
ZF0_RG	0.180	0.061	2.933	0.003
ZDUR	0.153	0.055	2.791	0.005

ZRELEN	0.233	0.066	3.552	0.000
HAPPY_T	-0.042	0.043	-0.986	0.324
AROU_T	0.561	0.069	8.087	0.000
**ZINT_M ON**				
**HAPPY_T**	**-0.084**	**0.029**	**-2.915**	**0.004**
AROU_T	0.766	0.027	28.572	0.000
ZINT_RG ON				
HAPPY_T	-0.064	0.046	-1.406	0.160
AROU_T	0.519	0.041	12.697	0.000
ZF0_05 ON				
HAPPY_T	-0.064	0.041	-1.571	0.116
AROU_T	0.684	0.030	22.449	0.000
ZF0_RG ON				
HAPPY_T	0.037	0.050	0.742	0.458
AROU_T	0.477	0.045	10.575	0.000
**ZDUR ON**				
**HAPPY_T**	**0.167**	**0.058**	**2.872**	**0.004**
AROU_T	-0.032	0.056	-0.563	0.573
**ZRELEN ON**				
**HAPPY_T**	**0.072**	**0.035**	**2.063**	**0.039**
AROU_T	-0.619	0.039	-15.675	0.000

INTONA WITH				
LOUD	0.108	0.066	1.635	0.102
PITCH	0.488	0.052	9.315	0.000
ROUGH	-0.254	0.055	-4.594	0.000
SPRATE	0.105	0.058	1.810	0.070
INSTAB	0.174	0.059	2.967	0.003
LOUD WITH				
PITCH	0.083	0.071	1.170	0.242
ROUGH	0.080	0.074	1.089	0.276
SPRATE	0.150	0.049	3.057	0.002
INSTAB	-0.166	0.060	-2.746	0.006
PITCH WITH				
ROUGH	-0.486	0.058	-8.445	0.000
SPRATE	0.226	0.053	4.278	0.000
INSTAB	0.300	0.060	5.027	0.000
ROUGH WITH				
SPRATE	-0.176	0.053	-3.311	0.001
INSTAB	0.132	0.061	2.167	0.030
SPRATE WITH				
INSTAB	-0.011	0.059	-0.189	0.850
ZINT_M WITH				

ZINT_RG	0.226	0.060	3.755	0.000
ZF0_05	0.198	0.058	3.389	0.001
ZF0_RG	0.315	0.052	6.008	0.000
ZDUR	-0.221	0.054	-4.051	0.000
ZRELEN	-0.562	0.053	-10.664	0.000
ZINT_RG WITH				
ZF0_05	0.097	0.060	1.600	0.110
ZF0_RG	0.154	0.060	2.552	0.011
ZDUR	0.289	0.059	4.933	0.000
ZRELEN	-0.097	0.055	-1.787	0.074
ZF0_05 WITH				
ZF0_RG	-0.175	0.064	-2.742	0.006
ZDUR	0.071	0.055	1.279	0.201
ZRELEN	-0.218	0.059	-3.682	0.000
ZF0_RG WITH				
ZDUR	0.094	0.061	1.529	0.126
ZRELEN	-0.222	0.053	-4.198	0.000
ZDUR WITH				
ZRELEN	0.076	0.056	1.351	0.177
Intercepts				
HAPPY_AS	0.757	0.099	7.665	0.000
INTONA	-0.254	0.075	-3.395	0.001

LOUD	-0.010	0.036	-0.271	0.787
PITCH	-0.197	0.072	-2.737	0.006
ROUGH	-0.099	0.106	-0.931	0.352
SPRATE	0.043	0.069	0.621	0.535
INSTAB	-0.536	0.087	-6.199	0.000
ZINT_M	-0.718	0.060	-11.879	0.000
ZINT_RG	-0.481	0.074	-6.467	0.000
ZF0_05	-0.648	0.055	-11.821	0.000
ZF0_RG	-0.499	0.080	-6.212	0.000
ZDUR	-0.065	0.097	-0.669	0.503
ZRELEN	0.577	0.074	7.810	0.000

Residual Variances

HAPPY_AS	0.381	0.031	12.299	0.000
INTONA	0.318	0.032	9.976	0.000
LOUD	0.083	0.009	9.285	0.000
PITCH	0.330	0.034	9.746	0.000
ROUGH	0.853	0.038	22.322	0.000
SPRATE	0.349	0.034	10.391	0.000
INSTAB	0.587	0.037	15.718	0.000
ZINT_M	0.406	0.040	10.158	0.000
ZINT_RG	0.727	0.042	17.391	0.000
ZF0_05	0.528	0.041	12.758	0.000
ZF0_RG	0.771	0.044	17.589	0.000
ZDUR	0.971	0.019	49.887	0.000
ZRELEN	0.612	0.048	12.765	0.000

R-SQUARE

Observed Variable	Estimate	S.E.	Est./S.E.	Two-Tailed P-Value
HAPPY_AS	0.619	0.031	19.950	0.000
INTONA	0.682	0.032	21.389	0.000
LOUD	0.917	0.009	102.516	0.000
PITCH	0.670	0.034	19.807	0.000
ROUGH	0.147	0.038	3.848	0.000
SPRATE	0.651	0.034	19.418	0.000
INSTAB	0.413	0.037	11.050	0.000
ZINT_M	0.594	0.040	14.863	0.000
ZINT_RG	0.273	0.042	6.535	0.000
ZF0_05	0.472	0.041	11.424	0.000
ZF0_RG	0.229	0.044	5.228	0.000
ZDUR	0.029	0.019	1.479	0.139
ZRELEN	0.388	0.048	8.086	0.000

QUALITY OF NUMERICAL RESULTS

Condition Number for the Information Matrix 0.199E-03

(ratio of smallest to largest eigenvalue)

TOTAL, TOTAL INDIRECT, SPECIFIC INDIRECT, AND DIRECT EFFECTS

Two-Tailed

	Estimate	S.E.	Est./S.E.	P-Value
Effects from HAPPY_T to HAPPY_AS				
Total	0.568	0.026	22.108	0.000
Total indirect	0.053	0.018	2.931	0.003

*Note.*All other expressed emotions are a reference category for happiness. Compared to other portrayals, portrayals that are in the happiness category display significantly less loudness (*b*=-.084), longer duration (*b*=-.167) and higher relative energy (*b*=.072). On the proximal side, high perceived intonation modulation (b=.185) and high perceived pitch (b=.016) contribute to a subjective judgment of happiness The amount of the total effect (.619) that is mediated through the acoustic measures and proximal percepts is relatively low (0.053). The relationship between the acoustic measures and the proximal percepts is very similar to the former analysis with happiness as reference category. The slight numerical differences are caused by the fact that the other emotions are not used as predictors for the proximal percepts in the current analysis.

Anger_t = expressed anger

Fear_t = expressed fear

Sad_t = expressed sadness

Arou_t = expressed arousal

anger_as = perceived anger (square-root-arcussinus transformed)

happy_as = perceived happiness (square-root-arcussinus transformed)

fear_as = perceived fear (square-root-arcussinus transformed)

sad_as = perceived sadness (square-root-arcussinus transformed)

intona = intonation

loud = loudness

pitch = pitch

rough = roughness

sprate = speech rate

instab = instability

zint_mean = intensity mean

zint_rg = intensity range

zf0_05 = F0 floor

zf0_rg = F0 range

zdur = duration

zrelen = relative energy

References

[S1] Juslin PN, Laukka P. Communication of emotions in vocal expression and music performance: different channels, same code? Psychol Bull. 2003;129: 770–814.

[S2] Bänziger T, With S, Kaiser, S. The face and voice of emotions: the expressions of emotions. In: Scherer KR, Bänziger T, Roesch EB, editors. Blueprint for affective computing: a source book. Oxford: Oxford University Press; 2010. pp. 85–104.

[S3] Bänziger T, Patel S, Scherer KR. The role of perceived voice and speech characteristics in vocal emotion communication. J Nonverbal Behav. 2014;38: 31–52.

[S4] Granqvist S. (1996). Enhancements to the visual analogue scale. Speech, Music and Hearing - Quaterly Progress and Status Report. 1996; 4: 61-65.

[S5] Bänziger T, Scherer KR. Introducing the Geneva multimodal emotion portrayal (GEMEP) corpus. In: Scherer KR, Bänziger T, Roesch EB, editors. Blueprint for affective computing: a source book. Oxford: Oxford University Press; 2010. pp. 271–294.
